# Cell therapies against brain tumors: Clinical development and emerging prospects

**DOI:** 10.1002/btm2.70018

**Published:** 2025-04-16

**Authors:** Tatsuya Fukuta, Suyog Shaha, Andres da Silva‐Candal, Zongmin Zhao, Samir Mitragotri

**Affiliations:** ^1^ John A. Paulson School of Engineering and Applied Sciences, Harvard University Allston Massachusetts USA; ^2^ Wyss Institute for Biologically Inspired Engineering Boston Massachusetts USA; ^3^ Present address: Department of Physical Pharmaceutics School of Pharmaceutical Sciences, Wakayama Medical University Wakayama Japan; ^4^ Present address: Neurovascular Diseases Laboratory, Neurology Service University Hospital Complex of A Coruña, Biomedical Research Institute A Coruña Spain; ^5^ Department of Pharmaceutical Sciences College of Pharmacy, University of Illinois Chicago Chicago Illinois USA

**Keywords:** brain tumors, cell engineering, cell therapy, clinical challenges, clinical trials, glioblastoma multiforme, immunotherapy

## Abstract

Malignant brain tumors, particularly glioblastoma multiforme (GBM), are aggressive and fatal cancers. The clinical efficacy of current standard‐of‐care treatments against brain tumors has been minimal, with no significant improvement over the past 30 years. Driven by the success of chimeric antigen receptor (CAR)‐T cells in the clinic for treating certain types of cancer, adoptive cell therapies have been of interest as a hopeful therapeutic modality for brain tumors. Clinical trials of GBM‐targeting cell therapies, including CAR‐T cells, have been initiated; however, none of them have been approved yet, and new challenges have emerged from the completed clinical trials. These issues are being addressed in ongoing clinical trials and recent preclinical research efforts. Herein, we present an overview of the clinical landscape of cell therapies against brain tumors. We analyze past and active 203 clinical trials focusing on cell therapies for brain tumors, discuss limitations for their clinical translation, and highlight emerging approaches to address these challenges. In addition, we review select preclinical studies that show promise to improve the therapeutic efficacy of therapeutic cells on brain tumors and discuss future prospects.

**Translational Impact Statement**

The clinical efficacy of standard‐of‐care therapies for malignant brain tumors has been very limited and has remained unchanged for decades. The use of living cells as therapies has gained significant interest for overcoming challenges faced by conventional therapeutics. However, several challenges have emerged in clinical trials pursuing this approach, and efforts to address these challenges are ongoing. This article provides an overview of the landscape in clinical trials for cell therapies against malignant brain tumors, discusses pressing clinical challenges, and highlights promising preclinical approaches.

## 1 INTRODUCTION

Although brain tumors account for only 1%–2% of all types of cancers, they are among the most deadly tumors.^1^ Malignant brain tumors can be categorized as primary brain tumors, which originate from intracranial tissues, and metastatic tumors, which are derived from tumors in other organs (e.g., lung, breast, and skin).^2^ Primary tumors include gliomas such as astrocytoma, ependymoma, and oligodendroglioma, which account for almost 80% of all brain tumor cases. Gliomas are historically classified as grade I and II, grade III, and IV (glioblastoma) by the World Health Organization (WHO).^3^ Among these, glioblastoma multiforme (GBM) accounts for over 50% of glioma cases and is highly aggressive, as characterized by its rapid proliferative and infiltrative properties. The current standard‐of‐care (SOC) therapy against GBM is composed of direct resection by surgery, followed by a combination of radiotherapy and chemotherapeutic treatment with temozolomide (TMZ). However, the overall median survival of GBM patients has been <15 months after definitive diagnosis. Even when most of the tumor mass is removed through the SOC therapy, fatal recurrence is almost inevitable, resulting in a 5‐year survival rate of <10%.^4^ The severe mortality rates have remained unchanged over the last 30 years. Various new therapies have been explored to address this dismal situation.^5^, ^6^ Recently, the FDA approval of Vorasidenib, a brain‐penetrant small molecule inhibitor targeting isocitrate dehydrogenase (IDH), marked the first new drug approval for brain cancer in decades.^7^

Immunotherapies, such as tumor vaccines, oncolytic viruses, immune checkpoint inhibitors, and cell therapy, have emerged as promising new treatments for brain tumors.^8^ For example, significant work has been performed to explore immune checkpoint inhibitors for GBM treatment, inspired by their success in various other solid tumors.^9^ In fact, the anti‐programmed cell death protein 1 (PD‐1) antibody (nivolumab) has demonstrated promising results for treating GBM in early‐phase clinical trials. However, phase III trials could not demonstrate clear clinical benefits, and no FDA‐approved immunotherapies for GBM exist to date.^10^ Such disappointing outcomes are often attributed to the severely immunosuppressed tumor microenvironment (TME) in GBM, characterized by both systemic and local immunosuppression, high intra‐ and intertumoral heterogeneity, highly infiltrative and invasive cell properties, and a low mutational burden.^11^ In addition, the blood–brain barrier (BBB) and blood‐tumor barrier (BTB) further hamper the delivery of therapeutic agents into brain tumors and necessitate high‐dose regimens.^12^ However, increasing the dosage of immunotherapies can lead to dose‐limiting off‐target effects and immune‐related adverse events.^13^ Therefore, overcoming the BBB and BTB to solve the problems of delivering therapeutic agents into the sites of brain tumors is critical for advancing immunotherapies in GBM treatment.

**FIGURE 1:**
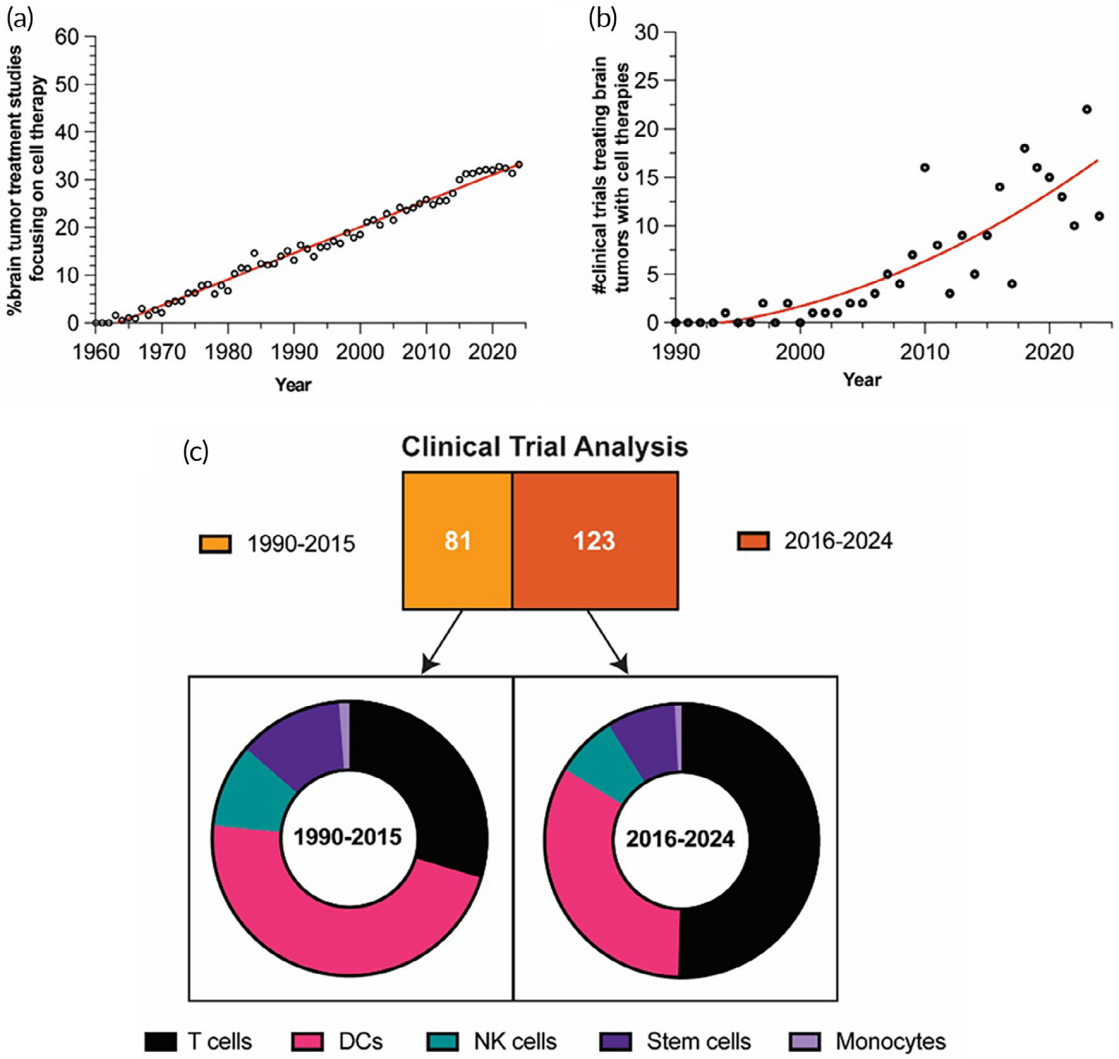
Clinical landscape of cell therapies for treating brain tumors. (a) The increase in the number of published literature (Identified from PubMed database) and (b) clinical trials (Identified from ClinicalTrials.gov database) involving treating brain tumors with cell therapies over the years. (c) The number of clinical trials identified for the clinical trial analysis from 1990 to 2015 and 2016 to 2024 and the relative proportion of various cell types used in these trials.

Cell therapies have significant potential to tackle these issues since they possess unique advantages over other conventional therapeutic modalities. Living cells have intrinsic abilities to efficiently cross biological barriers, including the BBB, and target specific cell types by responding to both systemic and local physical, chemical, or biological stimuli.^14^ In addition, certain types of cells have promising tropism for infiltrative brain tumor cells and the capability to eliminate them.^15^, ^16^ Further, genetic or non‐genetic modification of cells allows them to function more selectively and destroy tumor cells more effectively.^17^ The clinical success of T‐cells modified genetically with chimeric antigen receptor (CAR) in treating certain types of leukemia and lymphoma^18^ has motivated the genetic modification of therapeutic cells for the development of new therapies against GBM. As shown in Figure 1, the share of publications in the field of brain tumor treatment focusing on cell therapies, which started in 1963, has steadily increased year by year (Figure 1a). Notably, the number of publications on cell therapies for brain tumors significantly increased after the approval of CAR‐modified T cells (Kymriah) in 2017. Similarly, clinical trials employing therapeutic cells aiming to treat brain tumors started around 1993, and their number has continuously increased over the years (Figure 1b). T cells and dendritic cells (DCs) dominate the clinical landscape of cell types used in these trials, with a notable rise in the clinical trial number investigating the use of T cells after 2015 (Figure 1c). The identification of various upregulated antigens in brain tumors is driving the progress of antigen‐specific T cell therapies. Cancer vaccine approaches using DCs pulsed with the patient's tumor components (e.g., antigens and RNA) are also being explored to create personalized therapies for addressing the complex heterogeneity of GBM. Clinical trials involving other promising types of cells, such as natural killer (NK) cells and stem cells, are also ongoing, with some trials having progressed to phase 3. Cell therapy remains a promising candidate that could overcome the limited treatment options for brain tumors, especially GBM, though challenges and limitations from current clinical trials need to be addressed. Numerous preclinical studies of cell‐based therapies have been carried out to explore new strategies for overcoming these issues and improving poor therapeutic outcomes in brain tumor patients. However, a review that systematically focuses on diverse cell therapies for brain tumors from both clinical and recent preclinical perspectives is lacking. In the present review, we first discuss the history of developing cell therapies for treating GBM and then critically review clinical trials for each cell type used in brain tumor treatment. We have identified 203 clinical trials found on clinicaltrials.gov with the search on August 2024. Through analyzing trends in clinical trials, we highlight the limitations and challenges associated with the application of cell therapies in the clinic against malignant brain tumors. Moreover, we discuss recent promising preclinical studies focusing on new strategies to engineer cells as therapeutics or drug carriers to improve GBM treatment.

## 2 THE ORIGINS AND EARLY DEVELOPMENTS OF CELLULAR THERAPIES FOR BRAIN TUMORS

The emergence of cellular therapies has made a significant paradigm shift in brain tumor therapy. In this section, we review the early advances of cellular therapies for brain tumors, tracing their conceptual origins and the pivotal early efforts that have paved the way for current clinical advancements. One of the earliest and seminal cell therapy strategies for brain tumor treatment involved the application of lymphokine‐activated killer (LAK) cells. This earliest study with LAK cells in the treatment of malignant glioma was reported in April 1986.^19^ LAK cells were generated by activating peripheral blood lymphocytes (PBLs) of patients with brain tumors using recombinant interleukin‐2 (IL‐2) over a period ranging from 3 to 7 days. Activated LAK cells exhibited a remarkable ability to lyse both autologous and allogeneic GBM cells. Notably, PBLs from brain tumor patients without activation by IL‐2 could not significantly destroy GBM cells. Moreover, LAK cells derived from patients led to greater lysis of their own fresh tumor cells compared to the allogenic source. Importantly, these patient‐derived LAK cells did not lyse autologous normal PBLs, highlighting the specificity of the response. These findings underscored the potential of IL‐2‐induced LAK as an effective strategy for targeting GBM, opening a new avenue for cell therapies in neuro‐oncology.^20^ Takai et al.^21^ carried out in vitro and in vivo experiments using an experimental murine brain tumor model to examine the potential of LAK cell‐based treatment. This study showed that LAK cells exerted a lytic effect on both syngeneic tumor cells and allogeneic and xenogeneic tumor cells while showing no lytic activity against normal brain cells. In in vivo experiments using a gliosarcoma rat model induced by methylcholanthrene, LAK cells were adoptively transferred to the rats either intravenously or intratumorally. A significantly higher survival rate was seen in the LAK cell‐treated animals in comparison to the splenocyte‐treated or untreated control group, regardless of the administration route. Additionally, the area of necrotic foci was found to be greater in the tumors treated with LAK cells, indicating a potent antitumor response. In the same study, researchers administered radio‐labeled LAK cells intravenously to brain tumor‐bearing rats and found that adoptively transferred cells were able to infiltrate into the tumors. These seminal findings from this study supported the notion that adoptive transfer of LAK cells may be an effective approach for the treatment of malignant brain tumors.^21^ Their specific lytic activity against tumor cells enhanced survival rates, and tumor infiltration capabilities gave rise to the excitement for the potential therapeutic impact of cell therapy for brain tumors. Clinical studies using LAK cells, one of the first cell types utilized as therapies for treating brain tumors, were conducted on a cohort of nine patients with malignant glioma who had previously undergone various treatments.^22^ In this study, patients were given LAK cells, lymphokine IL‐2, or a combination of both LAK cells and IL‐2, which were directly administered into the resection cavity during surgery. LAK cells were autologous, and escalating doses were administered, ranging from 10^8^ to 10^11^ LAK cells and 10^4^ to 10^6^ U IL‐2. Notably, no indications of severe systemic or local neural toxicity and BBB damage were observed following the treatment, demonstrating the safety of the treatment. These findings demonstrated the feasibility of using LAK cells and IL‐2 to treat malignant glioma in a clinical setting. These early clinical observations were instrumental in initiating a new paradigm for GBM treatment using cell‐based therapies.

The findings from early preclinical and clinical studies with LAK cells provided foundational insights that paved the way for subsequent cell therapy strategies for GBM. Following these findings, tumor‐infiltrating lymphocytes (TILs) were identified as the next promising candidates for cell therapy. TILs demonstrated the potential for improved tumor targeting and enhanced eradication of tumor cells compared to LAKs.^23^ Initial research with TILs indicated the importance of immune cell infiltration into the TME and provided valuable insights into the potential mechanisms underlying TIL‐mediated anti‐tumor responses. Further preclinical and clinical studies expanded upon these initial findings to explore diverse aspects of TIL therapies.^24^ These studies focused on optimizing TIL isolation and expansion techniques, enhancing TIL infiltration into the tumor, augmenting TIL functionality, and combining TIL treatment with other modalities for improving therapeutic outcomes.^25^, ^26^, ^27^, ^28^ TIL therapies emerged as another effective tool for the development of more personalized treatment approaches for GBM treatment.

Subsequent studies and clinical trials have expanded upon these findings, exploring diverse cell therapies to advance the field. Clinical trials have continued to investigate the tolerability, efficacy, and long‐term outcomes of cell therapies for brain cancers. New advances in cell engineering, genetic modification techniques, and personalized treatment approaches have driven ongoing developments in the field. The clinical trajectory of cell therapy for GBM has witnessed remarkable progress over the years. While challenges remain, the continuous improvement in clinical trials offers hope for improved treatment outcomes in GBM patients. The clinical landscape and emerging trends in cell therapies for brain tumors were analyzed and discussed in the next sections.

## 3 CLINICAL TRIAL ANALYSIS FOR CELLULAR THERAPIES FOR BRAIN TUMORS

In this section, we categorized and discussed past and active clinical trials involving therapeutic cells for treating brain tumors. We searched clinical trials on the ClinicalTrials.gov database, with the keywords “brain tumor”, “glioma”, “glioblastoma”, and “brain metastases.” The data referenced in this section was obtained in August 2024.

### 3.1 T cell

**FIGURE 2:**
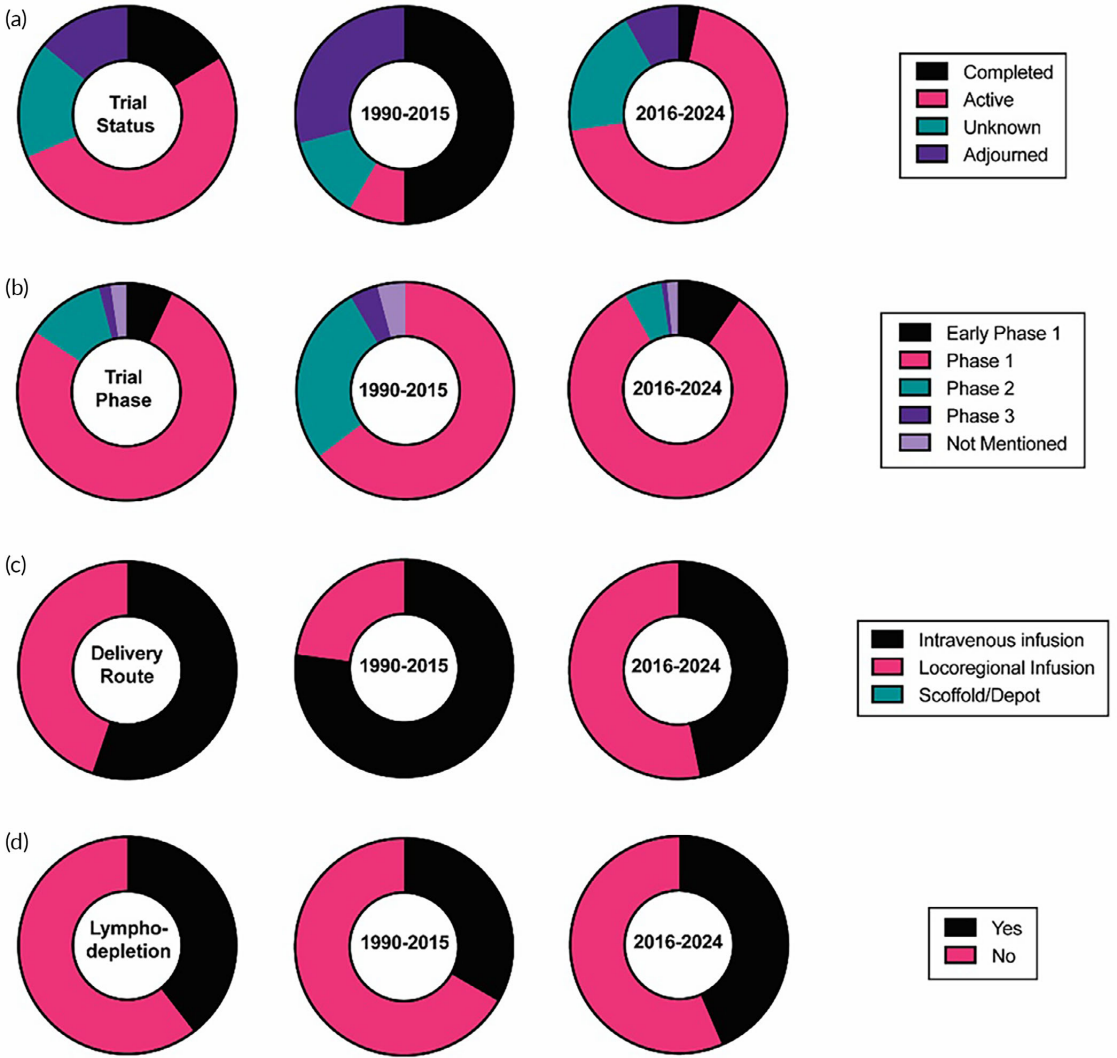
The current landscape of clinical trials using T cells for brain tumors. The distribution of trials based on (a) trial status, (b) trial phase, (c) delivery route, and (d) use of lymphodepletion is shown for 1990–2015, 2016–2024, and the overall period.

The recent progress of T‐cell therapy against diverse tumor types, especially in hematological cancers, generates enthusiasm for investigating their application in patients with brain tumors.^29^ The selectivity towards specific tumor antigen (s) offered by them is an emerging prospect for brain cancer management.^30^ With progress in understanding T cell activation and genetic engineering technology development, approaches to T cell transfer have evolved considerably over time from early minimally specific LAK cells to the newest precisely antigen‐specific CAR T cells.^31^ A total of 86 trials involving adoptive T cells have been registered for brain tumor therapy on the clinical trial database, with 52.3% of them having active status. The representative clinical trials using T cells are shown in Table 1. The number of these registered trials has been growing rapidly in the latest years, with 2023 being the year with the most registered trials. These show a large extent of ongoing efforts to translate T cell therapies for brain cancers. 91.3% of these trials use autologous cell sources for harvesting T cells. This high prevalence of autologous cell harvesting is similar to the overall pattern of current adoptive T cell therapies across all the spectrums.^32^ Autologous cells ensure prevention from the elimination of adoptively transferred T cells by host immunity, as well as diminish the risk of graft‐versus‐host toxicity.^17^ Notably, 84.3% of the trials (early phase 1, phase 1) have been in the early stages to examine the safety and feasibility of performing adoptive T cell transfer in patients with brain tumors. Only 15.7% of trials (Phase 2, Phase 2/3, and Phase 3) have moved to late‐stage large‐scale studies for evaluating the therapeutic efficacy. There is still a long way to go to establish promising benefits of T cell‐based therapies for brain cancer patients. To better understand how the trends with T cell therapies for brain tumors are shifting, we thought to separately analyze trials started from 1990 to 2015 as one group while trials started after 2015 till today as another group. 2015 marked the year when one of the seminal reports of success from clinical trials employing T cells for hematopoietic tumors was published.^33^ Additionally, 92% of trials registered between 1990 and 2015 for T cell therapies in brain tumors have ended, while 97% of the similar trials registered after 2015 are active (Figure 2a). Thus, by analyzing trials registered in 1990–2015 and 2015–present separately, we expected to find some emerging learnings and shifting trends. The 1990–2015 time interval accounts for 28% of trials, while 2015‐present interval accounts for the remaining 72% of trials. Unsurprisingly, the majority of trials in both time intervals (92% in the 1990–2015 interval and 88% in 2015‐present interval) have been early‐stage small trials with an average of 30 patients' enrollment, strongly suggesting that the clinical landscape of T cell therapies is still at its infancy even after a couple of decades of development (Figure 2b).

**TABLE 1:** Representative clinical trials for brain tumor therapies using T cells.

Start date (month/yy)	Clinical ID	Phase	Mechanism of action	T cell type	Target	Modification	Source	Administration	Combination
Aug‐1997	NCT00003185	2	Multiclonal population generated with tumor cell vaccination	Multiclonal‐selected	Tumor‐associated antigens	In vivo vaccination	Autologous	Intravenous	Lymphodepletion + Whole tumor cell vaccination
Feb‐2002	NCT00730613	1	MHC‐independent, surface target‐specific cytolysis	CAR‐transduced	IL13Rα2	Ex vivo Genetic Engineering	Autologous	Intracranial	No mention (NM)
Sep‐2008	NCT00693095	1	Polyfunctional anti‐tumor immunity generation	Monoclonal‐selected	CMV	Cellular Adjuvant with DC vaccine	Autologous	Intravenous	Lymphodepletion + DC vaccination
Oct‐2010	NCT01109095	1	CAR modification of virus‐specific T cell for bi‐dimensional response	CAR‐transduced	HER2 and CMV	Ex vivo Genetic Engineering of virus‐specific T cells	Autologous	Intravenous	NM
May‐2012	NCT01454596	1 and 2	MHC‐independent, surface target‐specific cytolysis	CAR‐transduced	EGFRvIII	Ex vivo Genetic Engineering	Autologous	Intravenous	Lymphodepletion
Jan‐2013	NCT01801852	NM	CD1d molecule restricted anti‐tumor effector response	Invariant natural killer T (iNKT) cells	CD1d	No	Autologous	Intravenous	NM
Jul‐2016	NCT02937844	1	Anti‐PDL‐1 CAR mediated cytolysis	CAR‐transduced	PD‐L1	Ex vivo Genetic Engineering	Autologous	Intravenous	Lymphodepletion
Aug‐2016	NCT02774291	Early phase 1	MHC‐dependent, intracellular target‐specific cytolysis	TCR‐transduced	ESO	Ex vivo Genetic Engineering	Autologous	Intravenous	Lymphodepletion + IL2 cytokine
Jan‐2017	NCT03347097	Early phase 1	PD1 antibody secreting tumor‐infiltrated lymphocyte	Tumor infiltrated lymphocytes	Tumor‐associated antigens	Ex vivo Genetic Engineering	Autologous	Intravenous	Lymphodepletion
Mar‐2018	NCT03344250	1	Bispecific antibody mediated targeted killing	Effector CD3+ T cells	EGFR	Ex vivo bispecific antibody coating	Autologous	Intravenous	Lymphodepletion
Jul‐2018	NCT03500991	1	MHC‐independent, dual surface target‐specific cytolysis	CAR‐transduced	HER2 and EGFR	Ex vivo Genetic Engineering	Autologous	Intracranial	NM
Feb‐2020	NCT04165941	1	MHC‐independent phosphine antigen mediated cytolysis with synergistic chemotherapy	TMZ resistant gamma‐delta T cells	NM	Ex vivo Genetic Engineering	Autologous	Intracranial	TMZ chemotherapy co‐administration
Jan‐2022	NCT05459441	Early phase 1	Ex vivo activation with ReteroNectin for bi‐dimensional specific T cell generation	RetroNectin activate killer (RAK) cells	NM	No	Autologous	Intracranial	IL2 cytokine
May‐2023	NCT05768880	1	Mixture of four MHC‐independent, surface target‐specific cytolysis	CAR‐transduced	B7‐H3, EGFRt, HER2, and IL13Rα2	Ex vivo Genetic Engineering	Autologous	Intraventricular	NM
Aug‐2023	NCT05685004	2 and 3	Multiclonal population generated with tumor cell vaccination	Multiclonal‐selected	Tumor‐associated antigens	In vivo vaccination	Autologous	Intravenous	Lymphodepletion+ Whole tumor cell vaccination
Apr‐2024	NCT06396481	Early phase 1	MHC‐independent phosphine antigen mediated cytolysis	Gamma‐delta T cells	NM	No	Allogenic	Intraventricular	NM

Abbreviations: CMV, cytomegalovirus; NM, no mention; TCR, T‐cell receptor.

The administration route of T cells is emerging as one of the key parameters in brain tumor treatment, especially with the challenges related to the unique BBB.^34^ Even though 44.8% of all clinical trials till now use a locoregional infusion strategy compared to 55.2% of trials with systemic intravenous administration, there is a recognizable shift in the delivery route chosen in the recent trials (Figure 2c). In the 1990–2015 interval, 77.1% of registered trials were injecting T cells intravenously compared to only 22.9% injecting cells locoregionally in the vicinity of the brain tumor. In contrast, 53.2% of registered trials after 2015 have been designed with locoregional infusion strategies, thereby slightly dominating over the intravenous administration route. Given the issues regarding effective immune cell homing in the brain microenvironment, locoregional strategies offer a way to bypass hurdles encountered during trafficking from circulation through a series of barriers.^29^ These locoregional methods include direct intratumoral and intraventricular administration. Some strategies implant reservoir/catheter delivery devices such as the Ommaya reservoir (NCT05459441, NCT04077866) during the surgical resection of brain tumors. The Ommaya reservoir is implanted just below the scalp using a catheter that reaches into the tumor cavity or bed. This shift towards locoregional delivery is also supported by preclinical studies comparing various routes for the delivery of T cells in brain tumor therapies.^35^ A study done in a brain metastases model stemmed from orthotropic breast cancer demonstrated that local infusion of HER2‐CAR T cells showed superior efficacy compared with a 10‐fold higher dose of cells delivered through intravenous infusion.^36^ Another study in the orthotopic GBM model demonstrated significant long‐term survival with local infusion of IL13Rα2‐CAR T cells, while intravenous injection of cells provided no significant benefit over the control group of untransduced cells.^37^ These studies indicate improvement in the safety and effectiveness of T cell‐based therapies with locoregional delivery for treating brain tumors. Despite the current limited clinical experience showing favorability for locoregional infusion, the optimal delivery route should be different based on tumor spread, the target tumor antigen, and antigen expression in the normal tissues.^38^ Results from more clinical studies are awaited to understand how the locoregional delivery route stands from the consideration of both patient outcomes and the standard of living for various brain tumor cases. Interestingly, we also did not find any clinical trials that deliver T cells through a scaffold or in a depot, even though a depot delivering chemotherapy locally in the brain was approved by the FDA in 1996. Keeping cells viable and functionally active in a depot for sufficient time is a big clinical hurdle for the translation of such strategies.^35^

Lymphodepletion conditioning is another critical component of clinical studies for adoptive T cell transfer therapies. All currently FDA‐approved T cell therapy products use lymphodepletion as a preparative conditioning regimen. This process depletes endogenous lymphocytes, creating a niche for T cell engraftment and enhancing the persistence of adoptive T cells, particularly after intravenous infusion. Additionally, the lymphodepletion regimen reduces tumor cells, leading to the release of soluble immunomodulatory factors and reprogramming of the tumor microenvironment. These effects further improve T cell homing and long‐term survival, ultimately enhancing therapeutic efficacy.^39^ Chemotherapeutic agents are commonly used in lymphodepletion, a practice influenced by the pre‐conditioning paradigm established in hematopoietic stem cell transplantation (HSCT),^40^ though it is generally less aggressive than that used for HSCT. However, it is becoming increasingly evident that lymphodepletion conditioning can lead to broad toxicity across various body systems. Further, cytopenia and opportunistic infection also contribute to reduced quality of life for patients during the T cell therapy cycle. Strategies to eliminate the need for lymphodepletion or to make it more targeted are of growing interest in the field, especially for brain tumors.^41^ 39.5% of total trials for T cell therapies in brain tumor treatment have some form of lymphodepletion conditioning in their treatment protocols. 43.5% of trials after 2015 have lymphodepletion conditioning in their treatment protocol compared to 29.2% of trials in the 1990–2015 time interval, indicating increased interest in combining lymphodepletion with T cell therapies in brain tumor treatments (Figure 2d). This is especially driven by the success of the lymphodepletion procedure in the protocol for approved T cell therapies for hematopoietic tumors.^32^ The delivery route of T cell infusion seems to be a key consideration for the inclusion of lymphodepletion in the protocol. With the emergence of locoregional administration of cells directly at the brain tumor site, the use of systemic lymphodepletion is becoming less common. Only 20.8% of trials with locoregional infusion of T cells include a lymphodepletion regimen in their protocol, while 55.8% of trials with intravenous infusion of T cells include a lymphodepletion regimen in their protocol. The majority of them are chemotherapy‐based lymphodepletion regimens. Using clinically approved TMZ at a non‐standard dose‐intensified regimen for lymphodepletion is proposed to be one of the attractive strategies, as the role of TMZ for brain tumor treatment along with lymphodepleting activity.^42^ Clinical trials (NCT00693095, NCT02664363) have been initiated to investigate this hypothesis. The lymphodepletion strategy requires further clinical validation for brain tumor treatments with intravenously delivered T cells, as the studies thus far have shown inconsistent results.^29^ As more clinical trials are being designed with locoregional T cell infusion for treating brain tumors, there is an increasing need to clinically assess the role of systemic lymphodepletion in the treatment regimen with locoregional administration of T cells.

**FIGURE 3:**
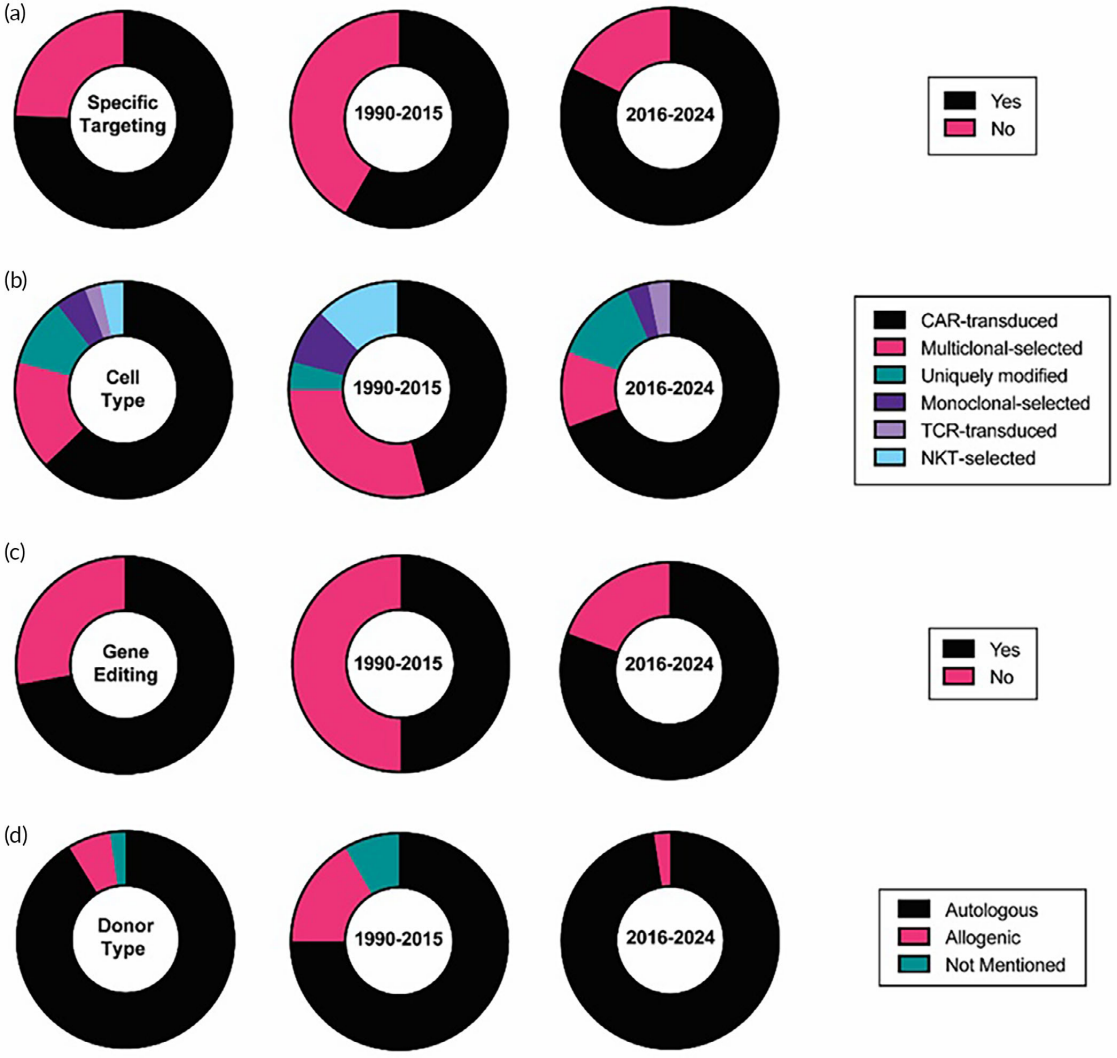
The current landscape of clinical trials using T cells regarding (a) use of specific targeting, (b) cell type, (c) use of gene editing, and (d) T cell donor type. The distribution of trials is shown for 1990–2015, 2016–2024, and the overall period, respectively.

Antigen‐specific killing is a crucial targeting mechanism for T cell therapies (Figure 3a). 75.6% of trials have a T cell engineered for a defined set of targets to utilize unique antigen‐specific killing mechanisms of T cells for controlled targeting of antigen‐expressing tumor cells. The initial LAK T cells used for brain tumor treatment did not have defined targeting. They were simply generated by ex vivo culture of the patient's PBLs in the presence of IL‐2 and were reintroduced into the patients as a therapy (NCT00331526, NCT00807027). LAK cells were not selected for tumor‐specific reactivity and contained a pool of multiclonal‐activated cells. Early studies found that LAK cells have the capability to lyse autologous tumor cells in vitro as well as selectively kill malignant brain tumors in vivo, prompting clinical investigation. The first exploratory clinical trial with LAK cell transfer in central nervous system (CNS) tumor patients was reported in 1986.^43^ However, with the emergence of more sophisticated engineering approaches, the clinical trials of LAK cells became less attractive in recent decades. The transfer of allogenic T cells for GBM is another earlier approach taken with hopes of generating host‐versus histocompatibility mismatch (NCT00002572, NCT01144247).^44^ However, the necessity for transplantation of allogenic hematopoietic stem cells (HSCs) as a part of treatment to avoid elimination by host immunity and the risk of graft‐versus‐host toxicity poses a significant hurdle. Hence, to minimize the non‐tumor effects, most T cell approaches today employ autologous T cell engineering for tumor antigen‐specific targeting. In fact, 82.2% of trials after 2015 have defined antigen targets compared to 58.3% of trials in the interval of 1990–2015 (Figure 3a).

Several engineering strategies have emerged for generating T cells against brain tumor antigens (Figure 3b). Some of the trials involve ex vivo activation of isolated PBMCs with autologous tumor cells/tissue.^45^, ^46^ Such tumor‐specific activation is also done in vivo by isolating CTLs from draining lymph nodes or the blood of patients vaccinated prior with autologous tumor cells or DCs. These isolated cells, the majority of which are already specific for tumor antigens, are subsequently simply expanded ex vivo and injected into the patients (NCT00003185, NCT01081223, NCT01290692, NCT03396575, NCT05685004). However, the availability of adequate patient tumor samples hampers the wide clinical use of vaccination‐based activation strategies.^17^ Another interesting strategy has been adoptive transfer with tumor‐infiltrating lymphocytes (TILs) isolated from brain tumors.^47^ To prevent TIL exhaustion in the TME of brain tumors, one preliminary study found that modifying them to secrete antibodies against the immune checkpoint receptor PD‐1 (NCT03347097) is safe and more effective. However, the utility of this approach is limited by the ability to isolate and expand TILs from brain tumor tissue.

To address the dearth of endogenous antigen‐specific T cells, genetic engineering has emerged as a promising strategy, with gene‐engineered CAR T cells leading the way in the development of brain tumors. 72.1% of T cell trials for brain tumors employ genetic engineering (Figure 3c). With the majority of approved T cell therapies employing genetic engineering, it is not a surprise that the genetic engineering of T cells for brain tumors is on the rise. 80.7% of trials after 2015 have genetically engineered T cells compared to 50% in the 1990–2015 interval. The CAR T cells currently dominate the clinical landscape for T cell‐based therapies against GBM. Our analysis found that 62.3% of T‐cell trials have been with CAR T cells. The cells are produced by genetically engineering patient‐derived T cells to express CAR proteins that are tumor‐specific antibodies or receptor ligands with an intracellular T cell signaling domain. With transcriptomics and proteomics profiling of brain tumors, more potential tumor‐associated targets have been identified and moved into clinical trials as targets for CAR T therapy.^48^ The tumor antigen candidate chosen should have negligibly low expression in the healthy brain but should have overexpression on malignant tumor cells. The most targeted GBM antigen is the mutant epidermal growth factor receptor (EGFR), which is upregulated in over 50% of GBMs. 30.7% of trials using CAR T cells for brain tumors target mutant EGFR. A study of dose escalation (NCT01454596) with CAR T targeting mutant EGFR in EGFRvIII+ GBM found that intravenously injected CAR T cells with systemic IL‐2 cytokine were well‐tolerated till dose levels of 100 million cells without evident off‐tumor targeting of EGFR. In another study, a single dose of EGFRvIII CAR T cells was intravenously administered into 10 patients having recurrent EGFRvIII+ GBM (NCT02209376). Molecular profiling and histopathology analysis of the tumors from these patients demonstrated the CAR T cell trafficking to brain tumors.^49^ There was a decreased level of EGFRvIII expression in tumors, indicating a reduction of EGFRvIII‐positive tumors. However, the residual tumor had enhanced expression of immune suppressive markers such as indoleamine 2,3‐dioxygenase 1 (IDO1), programmed cell death ligand 1 (PD‐L1), IL‐10, and a strikingly high immune suppressive cell infiltration including regulatory T cells (Tregs). These immunosuppressive responses following CAR T cell treatment suggest that combination with blockage of immune checkpoints might synergistically work with EGFRvIII‐CAR T therapies. One clinical trial is currently ongoing in this direction (NCT03726515). The unstable expression levels of mutant EGFR have been reported throughout tumor development, increasing the risk of escape with antigen loss for tumors targeted with EGFR CAR T cells. It exhibits a significant challenge for achieving a long‐term response in GBM patients with EGFR as a single target.

Besides mutant EGFR, IL13Rα2 is another popular antigen candidate that is overexpressed on brain tumor cells compared to healthy brain tissue. Its expression increases more with the development of tumors. It was an early target for CAR T‐cell therapy in brain cancer (NCT00730613). In the early days, two clinical trials were started to investigate the effect of the first generation of IL13Rα2 CAR T cells using locoregional tumoral delivery for GBM. One trial involved an autologous cell source (NCT00730613), while the other had allogenic CAR T cells (NCT01082926). There was no dose‐limiting toxicity at a dose of 100 million CAR T cells. Further, these trials demonstrated their limited persistence with the short‐lived anti‐tumor effect.^50^ To improve the persistence post‐injection, second‐generation CAR T cells were developed with an additional co‐stimulatory domain and optimized space domain. These cells gave an approximately 10‐fold enhancement in anti‐tumor effect in comparison to first‐generation cells.^51^ The ongoing clinical trial (NCT02208362) with these CAR T cells has established the absence of dose‐limiting toxicity after a locoregional infusion into brain tumors. One patient in this trial remarkably had complete remission. Recurrent tumor cells appeared outside the brain with low IL13Rα2 expression levels. This antigen escapes likely, causing tumor recurrence. Several other trials investigating the effects of CAR T cells targeting IL13Rα2 are underway in certain types of brain tumors including GBM, medulloblastoma, and ependymoma (NCT04510051, NCT04661384, NCT04003649).

Human epidermal growth factor receptor 2 (HER2) is another widely used target in CAR T cell therapy. HER2 is highly abundant in brain tumors with low levels in normal tissue, including healthy brain tissue.^52^ Compared to the earlier two candidate antigens, EGFRvIII and IL13Rα2, HER2 has slightly more prevalence in healthy tissue; thereby, the higher risk of off‐target effect exists. The first patient treated in the clinical setup with CAR T cells targeting HER2 died because of a cytokine storm resulting from off‐target toxicity in pulmonary epithelium.^53^ The adverse effects were mitigated with a newer design of CARs, where the trastuzumab‐based antigen‐binding ectodomain was replaced with an FRP5‐based ectodomain. Additionally, the persistence was enhanced by utilizing CMV virus‐specific T cells for genetic engineering. These modifications aimed to improve targeted antitumor activity through the CAR while ensuring sustained function by receiving costimulation from latent virus antigens. Serious adverse events were not observed in the clinical trial (NCT01109095), which evaluated the safety of this updated version of HER2 CAR T cell therapy in GBM.^54^ This trial also showed a prolongation of median survival in patients. Inspired by the outcomes from this initial trial, many trials are underway to evaluate effects in HER2‐positive malignant glioma (NCT03389230, NCT02442297), brain metastasis of HER2‐positive malignant glioma (NCT03696030), and ependymoma (NCT04903080).

Another intriguing target that has gained attraction over recent years is the human cytomegalovirus (CMV) antigen present in brain tumors. Several studies found CMV antigen in human glioma while it is absent in normal tissues, including surrounding healthy brain tissue.^55^ One strategy is using autologous CMV‐specific T cells that are already present in peripheral blood. These cells are isolated from the patient's peripheral blood and expanded ex vivo. A clinical trial (NCT02661282) with their adoptive transfer indicated the treatment was well tolerated. However, this trial failed to exhibit anti‐tumor benefits as the effector function of the injected T cells was greatly suppressed.^56^ A trial (NCT00990496) with an allogenic source of CMV‐specific T cells was also started, although it was discontinued because of low accrual. In another interesting study, a combination of vaccination with CMV pp65 RNA‐loaded dendritic cells and CMV‐specific T cells was clinically evaluated (NCT00693095) to increase the frequency of polyfunctional T cells. There was a significant increase in the total number of polyfunctional effector CMV‐specific T cells in patients who received a combination. Such increases have been correlated with the overall survival.^57^ A larger randomized study will be needed to evaluate the promise shown in this study. Additionally, genetic engineering to develop CMV‐specific CAR T cells has also been an attractive platform. Clinical trials (NCT01205334, NCT01109095) have been undertaken to evaluate the applicability of this approach. Additionally, a phase 1 trial with systemic administration of CAR T cells specific for CMV as well as HER2 was found to be safe with a durable clinical benefit in ~38% of patients.^58^ The CMV specificity enabled these bispecific CAR T cells to be more active since they reacted with the virus as well as with tumor cells.

While the above‐mentioned antigen targets show promising benefits in brain tumor treatment, the therapeutic effects have not been long‐lasting.^48^ The long remission is not achieved due to challenges posed by target antigen loss, high brain tumor heterogeneity, and immunosuppressive TME. These challenges are being addressed by targeting alternative antigens, developing CAR T cells simultaneously targeting multiple receptors, and combining therapies that would enable antigen spread as well as overcome immunosuppression.^29^ The quest for a target antigen that is homogenously expressed on brain tumor cells without any expression anywhere else is ongoing. Some newly discovered antigens have shown great promise in preclinical studies.^48^ Few of them have moved for clinical evaluation. This includes B7‐H3 (NCT04077866, NCT05835687, NCT04385173, NCT05366179, NCT05474378, NCT05241392), EphA2 (NCT03423992), GD2 (NCT04099797, NCT04196413, NCT05298995, NCT05544526), MUC1 (NCT02617134), CD147 (NCT04045847), matrix metalloproteinase‐2 (MMP2; NCT04214392, NCT05627323), NKG2D (NCT04270461, NCT04550663, NCT05131763), CD70 (NCT05353530), IL7Ra (NCT05577091). All of these trials involving CAR T cells targeting new antigens are ongoing, and none of them has reached the endpoint. It will be interesting to see if any of the targets work provides better therapeutic benefit than mutant EGFR, IL13R α2, and HER2.

The preliminary reports suggest limited therapeutic impact in the clinical setting by targeting a single antigen.^38^ Thus, simultaneous targeting of multiple antigens with Bi/Tri/Quad specific CAR T cells is another avenue sought to address antigen escape. Antigen escape happens due to tumor heterogeneity and antigen loss. Brain tumors, being highly heterogeneous, likely consist of tumor cell clones without targeted antigen expression. Further, tumor cells may downregulate a single target antigen during treatment. Tumors recur after monovalent CAR T cell therapy with such target antigen downregulation or antigen‐negative clone emergence.^49^, ^59^ By targeting multiple different antigens simultaneously, the likelihood of antigen escape can be reduced, leading to long‐term remission. Such multi‐antigen targeting is either done by engineering multivalent CAR T cells or pooling different single‐targeting CAR T cells.^60^ In vivo preclinical studies suggested that multivalent CAR T cells show stronger anti‐tumor effects than mixing single‐targeting CAR T cells. Multivalent CAR T cells have been developed with either the expression of multiple discrete receptors, a single receptor with multiple targeting‐binding domains, or tandem receptors.^61^ HER2 and IL13Rα2 targeting bispecific CAR T cells showed therapeutic benefits in orthotropic murine glioma studies with enhanced anti‐tumor response and avoidance of antigen loss.^62^ This study developed a mathematical model to demonstrate that targeting HER2 and IL13Rα2 simultaneously could cover all primary tumor cells present in the brain tumor for targeted killing with CAR T cells. In another study, tri‐specific CAR T cell therapy targeting EphA2, HER2, and IL13Rα2 in GBM exhibited superior anti‐tumor benefits compared with single or dual antigen targeting. Further, their mathematical modeling indicated that these tri‐specific CAR T cells could destroy almost all tumor cells in 15 brain tumor patients with expression of variable antigens. Currently, a Phase 1 trial investigating the safety and feasibility of mutant EFGR and IL13 Rα2 targeting bispecific CAR T cells is underway for patients with GBM (NCT05168423). In another interesting development, a first phase 1 trial investigating safety and efficacy after locoregional delivery of B7H3, HER2, EGFR806, and IL‐13Rα2 quad‐targeting CAR T has been recently commenced (NCT05768880).^63^ Quad CAR T cells are produced by simultaneous transduction of patients' T cells with four different vectors. The Quad T cells are highly effective preclinically in diffuse midline gliomas compared to conventional CAR T cell approaches. An in vivo study using an orthotropic mouse brain tumor model demonstrated significant survival benefit. It will be interesting to see if these Quad‐CAR T cells could offer a curative response within tolerable limits after locoregional delivery.

In addition to genetically engineered CAR T cells, genetic modification of T cells with TCR has also moved to clinical trials for brain tumors. While current CAR T cells, which dominate the clinical landscape, are capable of recognizing only surface targets, TCR T cells open new avenues with the ability to recognize MHC complex‐bound peptides derived from intracellular proteins.^32^ These approaches use human leukocyte antigen (HLA)‐matched T cells to avoid any alloreactivity. The use of TCR‐transduced autologous peripheral blood lymphocytes is examined to treat patients with metastatic tumors in the brain (NCT02774291). The TCR‐transduced cells in this strategy target the Anti‐ESO antigen, which is an immunogenic peptide generated from the processing of cancer‐testis antigen.^64^ Another clinical trial (NCT05478837) is investigating genetically modified TCR‐T cells that target the H3.3K27M epitope in patients with diffuse midline glioma (DMG).^65^ However, there is some skepticism about the expression of the mutant H3.3K27M epitope in DMG cells, which might render the approach of using Anti‐H3.3K27M TCR‐expressing T‐cells ineffective.

The use of γδT cells, which are emerging to be another interesting T‐cell type for cancer therapy, has moved into clinical investigation.^66^ In humans, these cells represent 1–10% of the total CD3+ T‐cell population. They serve as a bridge between innate and adaptive immune systems. Activated γδT cells can elicit an anti‐tumor response in an HLA‐independent manner by responding to cellular stress markers and antibody‐opsonized target cells.^67^ High γδT cell presence in tumors has been associated with improved patient outcomes.^68^ The γδT cell transfer has been demonstrated to extend survival with slow tumor progression in mice with orthotropic glioma xenograft.^69^ In one interesting strategy, TMZ, a clinically approved chemo‐drug to treat brain tumors, was combined with γδT cell transfer.^70^ TMZ treatment just increases the stress‐associated markers on glioma cells with resistance to TMZ‐induced killing. Co‐delivery of γδT cells renders these resistant cells vulnerable to γδT cell killing. The transferred γδT cells needed genetic modification to be resistant to the toxic effects of TMZ. These TMZ‐resistant γδT cells displayed robust anti‐tumor activity against the GBM cell line by combination with high concentration of TMZ. Phase 1 and 2 clinical studies are ongoing in GBM patients to evaluate the safety and efficacy of the combination of TMZ with TMZ‐resistant γδT cells (NCT05664243, NCT04165941). It will be interesting to see whether these γδT cells emerge as a potential alternative to conventional αβT cell therapies for brain tumors.

In addition to coming up with new strategies to engineer and source T cells, combination with other therapeutic modalities is also a prospective approach to improve the cold brain tumor environment and achieve adoptive T cell effective activity.^30^ Combination with an immune checkpoint inhibitor and T cell treatment is one such promising strategy. Clinical studies with immune checkpoint inhibitors such as CTLA‐4 and PD‐1 have been shown to modify the TME in glioma patients.^71^ This alteration with PD‐1 blocking could synergize with T cell treatment and lengthen patient survival. Indeed, combining these checkpoint inhibitors has been demonstrated to increase the anti‐tumor efficacy of CAR T cells on brain tumors in preclinical models.^72^ The combination of EGFRvIII CAR T cells with an anti‐PD‐1 mAb (pembrolizumab) has been explored in a clinical trial NCT03726515. The study is completed, but the results are awaiting. Similarly, the combination of IL13Ra2 CAR T cells with ipilimumab (anti‐CTLA‐4) and nivolumab (anti‐PD‐1) is currently being investigated for treating GBM (NCT04003649). The clinical trial NCT03294954 is also underway, where GD2‐specific CAR T cells that express IL15 are being investigated for brain tumor treatment. The IL15 expression is aimed at providing in‐situ cytokine support for improving the persistence and antitumor activity of CAR T cells.^30^ A combination with IL2 cytokine has also been employed in one of the clinical trials to support CAR T cell activity (NCT01454596). The combination with vaccination‐based strategies is also being evaluated. Many trials (NCT00003185, NCT01326104, NCT01081223, NCT01290692, NCT03334305, NCT03396575, NCT04837547, NCT05685004) have been performed by vaccinating with peptides or peptide‐pulsed DCs before the initiation of T cell therapy to generate and expand the tumor‐specific T cell repertoire. This diversified library of T cells available in the blood is then isolated from the blood, expanded ex vivo, and then injected as a therapy. One interesting phase 1 study injected CMV‐specific T cells with CMV‐pulsed DCs into GBM patients with the aim of antigen spreading (NCT00693095). It was found that combination with vaccination significantly increased the polyfunctional CD8+ T cell frequencies.^57^ This supports further evaluation of this approach in a large, randomized study for evaluating effective anti‐tumor response and overall survival improvement.

91.3% of T cells employed in the clinical trials are autologous (Figure 3d). The application of allogenic T cells for brain tumors is becoming less prominent over the period. 75% of trials during the 1990 to 2015 period were using autologous cells, but after 2015, 98% of trials have been started with autologous sources. This dominance of using autologous sources is also seen in trials for other tumor types. The graft‐versus‐host disease (GVHD) resulting from strong MHC‐dependent restriction exhibited by the T cells in their killing is likely the reason for the allogenic source not being preferred.^32^

Clinical experience with adoptive T cells for brain tumors until now has provided valuable insights to help in shaping recent trials. Locoregional administration, inclusion of lymphodepletion in the dosing regimen, well‐defined multiple antigen targeting, genetic engineering of T cells, especially for CAR T production, and autologous cell sourcing are key aspects that are increasingly becoming popular in T cell clinical trial design. Initial indications warrant the need for the therapies to act simultaneously or in series through multiple pathways to overcome the different obstacles posed by brain tumors. It will also be critical for strategies that have already been found safe to be incorporated in future trial designs to shed more light on underlying resistance and their application for generating therapeutic responses. This information will enable a path forward with better designs of T cell regimens and combinations to overcome limitations for brain tumor treatment and translate promising T cell therapies for brain tumors.

### 3.2 Dendritic cells

**FIGURE 4:**
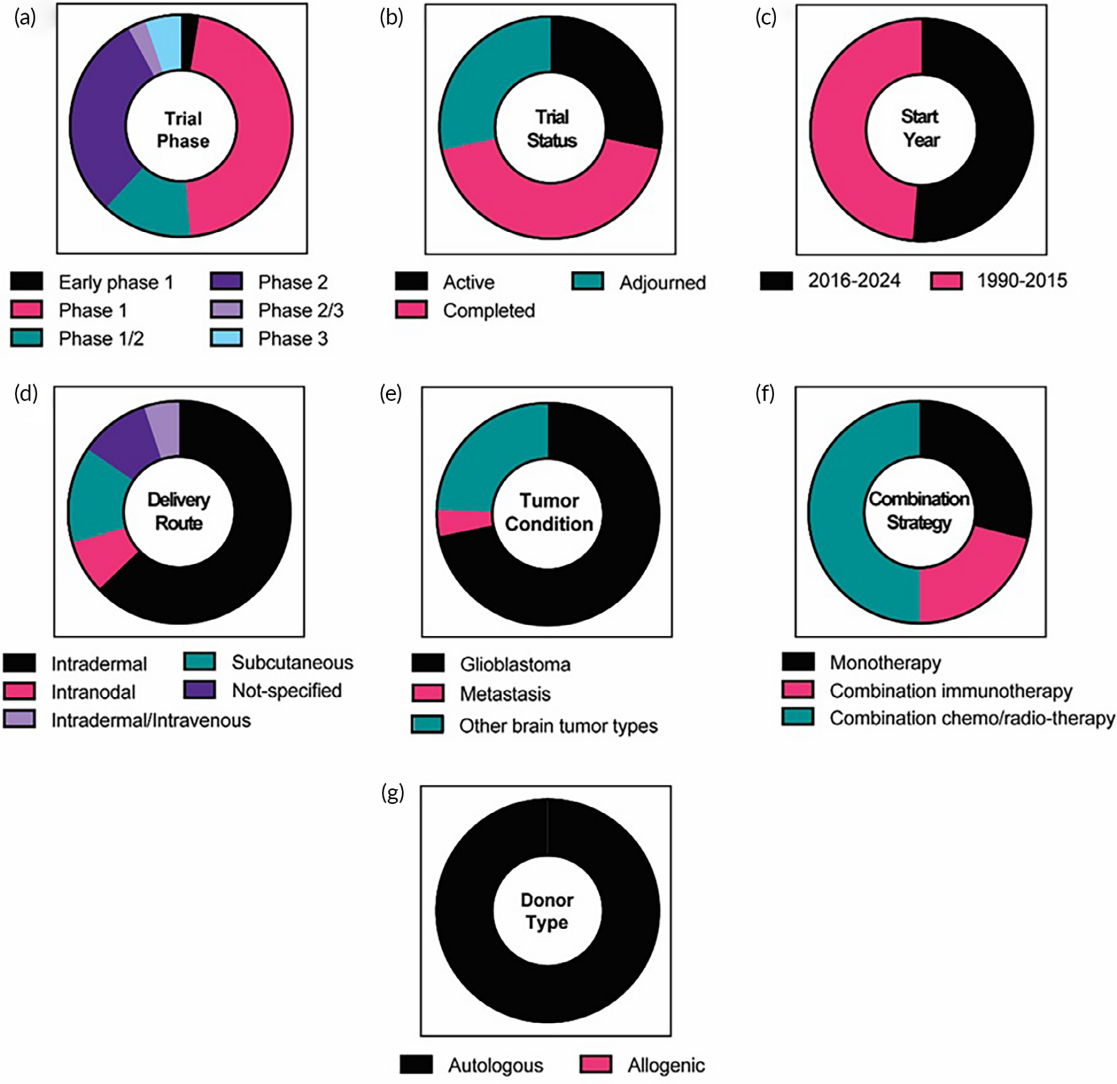
The clinical landscape of DC‐based clinical trials. A total of 79 clinical trials were identified and analyzed according to (a) trial phase, (b) trial status, (c) start year, (d) delivery route, (e) tumor conditions (GBM, metastasis, or other types of brain tumors), (f) use of combination strategies, and (g) donor type.

DCs are the main antigen‐presenting cells (APCs) involved in the crosstalk of the innate and adaptive immune systems to induce immune responses against tumors. Numerous preclinical studies revealed that treatment with DC vaccines induces tumor‐specific cytotoxic T‐cell responses in association with the infiltration of T cells into GBM tissue, which allows for the suppression of tumor growth, the extension of the survival rate, and long‐lasting antitumoral memory.^73^ In addition, many clinical trials have been started in brain tumor patients, and the feasibility and safety of the DC vaccines have been shown. At present, there are a total of 79 clinical trials utilizing DCs for brain tumor therapy, with 29.2% (23 trials) of them having active status. The DC‐based trials account for 39% of overall trials using therapeutic cells (Figure 4). Among all trials utilizing therapeutic cells, DC‐based therapy is the second most sought‐after cell type following T‐cell‐based therapy. Representative examples of trials with DCs are presented in Table 2.

**TABLE 2:** Examples of active/completed clinical trials for DC vaccines for brain tumor therapy.

Start date (month/yy)	Status (phase)	Clinical ID	Tumor condition	Cellular source	Priming materials	Injection route	Combined treatment
Jan‐2024	Not yet recruiting (Phase 3)	NCT05100641	Primary GBM	Autologous	Autologous tumor antigens	Subcutaneous	NM
Oct‐2010	Active, not yet recruiting (Phase 2)	NCT01204684	Glioma, GBM, Anaplastic astrocytoma/astrooligodendroglioma	Autologous	Autologous tumor antigens	Intradermal	Resiquimod Poly ICLC
Mar‐2018	Recruiting (Phase 2)	NCT03395587	GBM	Autologous	Autologous tumor lysate	Intradermal	TMZ Fractionated radiotherapy
Mar‐2021	Recruiting (Phase 2)	NCT04523688	GBM	Autologous	Autologous tumor homogenate	Intradermal	TMZ
Feb‐2018	Recruiting (Phase 1/2)	NCT03879512	Childhood GBM	Autologous	Autologous tumor lysate	Intradermal	Metronomic cyclophosphamide, Nivolumab, Ipilimumab
Jan‐2020	Recruiting (Phase 1)	NCT04201873	Recurrent GBM	Autologous	Autologous tumor lysate	Intradermal	Pembrolizumab (Neoadjuvant treatment) Poly ICLC
Aug‐2020	Recruiting (Phase 2)	NCT03688178	GBM	Autologous	Human CMV pp65‐LAMP mRNA	Intradermal (Bilaterally at the groin site)	Varlilumab Td TMZ
Sep‐2021	Not yet recruiting (Phase 1/2)	NCT04911621	High grade glioma Diffuse intrinsic pontine glioma	Autologous	WT1 mRNA	Intradermal	TMZ Radiotherapy
Dec‐2015	Recruiting (Phase 1/2)	NCT02649582	GBM	Autologous	WT1 mRNA	Intradermal	TMZ Radiotherapy
Sep‐2010	Active, not recruiting (Phase 2)	NCT01326104	Medulloblastoma Neuroectodermal tumor	Autologous	TTRNA	Intradermal	TTRNA‐xALT
Jul‐2018	Recruiting (Phase 1)	NCT03396575	Diffuse intrinsic pontine glioma Brain stem glioma	Autologous	TTRNA	Intradermal	TTRNA‐xALT GM‐CSF, Td TMZ, Autologous HSCs, Cyclophosphamide/Fludarabine
May‐2018	Active, not recruiting (Phase 1)	NCT03334305	Malignant glioma High grade glioma	Autologous	TTRNA	Intradermal	TTRNA‐xALT GM‐CSF, TMZ, Td, Autologous HSCs
Sep‐2021	Recruiting (Phase 1)	NCT04837547	Neuroblastoma Diffuse intrinsic pontine glioma	Autologous	TTRNA	Intradermal	TTRNA‐xALT
Dec‐2024	Not yet recruiting (Phase 1)	NCT05457959	Diffuse Hemispheric Glioma, H3 G34‐Mutant	Autologous	Tumor peptide	Intradermal	Nivolumab, Ipilimumab, Poly ICLC
Jul‐2021	Recruiting (Phase 1)	NCT04968366	GBM	Autologous	Multiple tumor neoantigen peptides	Intradermal	TMZ
Apr‐2018	Active, not recruiting (Phase 2/3)	NCT03548571	GBM	Autologous	mRNA from autologous tumor stem cells, survivin, and hTERT	Intradermal	TMZ
Dec‐2022	Recruiting (Phase 2)	NCT04348747	Brain metastasis (from triple negative breast cancer)	Autologous	Tumor‐associated antigens HER2 and HER3	Intradermal	Pembrolizumab
May‐2006	Completed (Phase 2)	NCT00323115	GBM	Autologous	Autologous tumor lysate	Intranodal (Cervical lymph node)	TMZ Radiotherapy

Abbreviations: NM, no mention; Poly ICLC, polyinosinic‐polycytidylic acid; TTRNA‐xALT, tumor total RNA‐tumor specific ex vivo expanded autologous lymphocyte transfer.

The details of the phase (Figure 4a) and status (Figure 4b) of the trials using DCs for brain tumor treatment are shown in Figure 4. 29.1% (23 trials), 43.0% (34 trials), and 27.9% (22 trials) of the trials are in the status of active, completed, and adjourned, respectively. 88.6% of the trials have been in the early phase of clinical trials. Notably, there is only one phase 2/3 and one phase 3 trial with active status. Interestingly, the number of trials started before and after 2015, which marks a key year for T‐cell therapy in cancer treatment, is almost the same (Figure 4c), suggesting DCs are still the cells of interest in the clinics.

For patients with brain tumors, 68% of the trials are performed by administration of DC vaccines through the intradermal route, while subcutaneous (7.1%) and intranodal (7.1%) are other preferred routes. Unsurprisingly, intravenous injections are the least favorable route for DC vaccines, owing to their mechanism of action involving entry into lymph nodes through the lymphatic system. In certain trials (NCT04888611, NCT04388033, and NCT00323115), the sites of intradermal or intranodal injection were specified as close to or into the cervical lymph nodes (CLNs), respectively. Deep CLNs are the main drainage lymph nodes of the CNS, while mandibular CLNs are involved in antigen sampling in the CNS.^74^ In addition, meningeal lymphatic vessels (MLVs), located both basally and dorsally beneath the skull, have recently been indicated to have a critical role in brain tumor drainage and immune response by allowing the trafficking of immune cells from the CNS into CLNs.^75^ The results of the phase 2 trial (NCT00323115) employing CLN injection of autologous tumor lysate‐DC vaccine combined with TMZ and radiotherapy have demonstrated them to be feasible and safe in patients with GBM and to have the capability to elicit tumor‐specific immune responses.^76^ The results of the other two active trials employing dendritic/glioma cell fusion vaccine (Phase 1/2; NCT04388033) and DCs pulsed with GBM stem‐like cell antigens (Phase 2; NCT04888611), respectively, with injection to CLN will be interesting, although the status of these trials is unknown.

Ex vivo pretreatment of DCs is essential to induce a robust anti‐tumor immune response with a DC vaccine. Trials for patients with brain tumors employ three different methods. The most common method is the pretreatment of DCs with an autologous tumor antigen cocktail or lysate isolated from patients' tumor tissue during surgery or tumor cell lysate prepared by the primary culture of allogeneic tumor tissue. The most progressed active phase 3 trial utilizes autologous tumor antigen‐loaded DCs, a DC product named AV‐GBM‐1 (NCT05100641). The phase 2 study using AV‐GBM‐1 combined with radiation and TMZ for newly diagnosed GBM patients demonstrated that AV‐GBM‐1 can be reliably manufactured, well‐tolerated, and might increase median progression‐free survival.^77^ In another phase 3 trial (NCT00045968) utilizing autologous DCs pulsed with tumor lysate termed DCVax‐L, the combination of DCVax‐L and TMZ was shown to be well‐tolerated and induced a clinically meaningful and statistically significant increase in both patients with newly diagnosed and recurrent GBM.^78^ There are several advantages of using autologous tumor lysate for the DC vaccine. First, we can ensure that the vaccine treatment targets a broad range of antigens present in patients' tumor tissue regardless of the extreme heterogeneity of GBM. Second, targeting the full antigen repertoire can prevent the maturation of the patients' tumors, although their maturation has been observed in some cases.^79^ The second most common method for ex vivo DC pretreatment is loading (or pulsing) DCs with mRNA. Pulsing DCs with mRNA‐encoding defined tumor antigens is simple, effective, and reproducible since mRNA with already known sequences can be rapidly prepared in vitro. mRNA of CMV pp65‐lysosomal‐associated membrane protein (LAMP) and Wilms' tumor 1 (WT1) has been used in a lot of trials to prepare DC vaccines for brain tumors. Expression of proteins unique to human CMV, including the immunodominant protein pp65‐LAMP, has been found in most malignant gliomas but not in healthy glial tissues.^80^ The transcription factor WT1 is also found to be specific to GBM.^81^ The completed phase 1/2 trial reported that the DC vaccine pulsed with CMV pp65‐LAMP mRNA combined with adjuvant granulocyte‐macrophage colony‐stimulating factor (GM‐CSF) showed long‐term OS and PFS in GBM patients.^82^ Notably, four active trials have been utilizing total tumor RNA (TTRNA)‐loaded DCs as a novel platform (NCT01326104, NCT03396575, NCT03334305, NCT04837547). This platform aims to induce strong immune responses against diverse uncharacterized and patient‐specific antigens found on tumor cells. The advantage of using TTRNA is that abundant amounts of antigens can be obtained without the identification of specific tumor antigens for each patient. Further, the required mRNA content of tumor cells for the loading on DCs can be generated from even microscopic amounts of tumor tissue by amplifying TTRNA with techniques of PCR. The most advanced trial (phase 2/3, NCT03548571) with an mRNA‐pulsed DC vaccine utilizes DCs transfected with human telomerase (hTERT) and survivin mRNA from autologous GBM stem cells (GSCs). The increased activity of hTERT, as well as high expression of survivin, was found in GSCs.^83^ Phase 1/2 with this product has demonstrated that it can elicit a GSC‐specific immune response without severe adverse reactions and may extend recurrence‐free survival.^83^ Another method to prepare the DC vaccine is loading DCs with peptides from tumor antigens. The advantages of using peptide‐based DC vaccines are cost‐effective manufacturing, convenient production, and low risks of pathogen contamination. However, since intra‐ and inter‐tumor heterogeneities are one of the major problems for treating GBM using immunotherapies, identification of epitopes derived from GBM tumor antigens is a challenge to elicit continuous and strong immune responses in diverse patients when compared to the tumor lysate‐based approach, making this peptide loading approach the least favorable among others.

DC‐based brain tumor therapy is mostly applied to GBM among other brain tumor types, with only lesser applications in metastatic brain tumors (Figure 4e). The therapeutic mechanism of action is not only for GBM but also for other brain tumors, such as neuroblastoma, diffuse intrinsic pontine glioma (DIPG), and medulloblastoma. Other distinct aspects of the trials using the DC vaccine are a combination with other therapeutic modalities (Figure 4f) such as immunostimulatory adjuvants (e.g., resiquimod and tetanus‐diphtheria (Td) toxoid) and cytokines (e.g., GM‐CSF and IL‐12) that promote DC trafficking to the lymph nodes, immune checkpoint inhibition with anti‐PD‐1 (nivolumab, camrelizumab, permbrolizumab) and anti‐CTLA‐4 (ipilimumab) that remove the brakes on generating T‐cell responses by the DC vaccine. As a new treatment strategy, a recent phase 1/2 trial (NCT03879512) performed a short cycle of metronomic cyclophosphamide treatment that can deplete Tregs without inducing general leukopenia.^84^ The Treg depletion lowered systemic immunosuppression in high‐grade glioma patients and enhanced the therapeutic efficacy of DC vaccines. More specific Treg inhibition by a monoclonal antibody against CD27 (varlilumab), which depletes Treg without impairing the effector T‐cell activity, has also been employed in a phase 2 study to enhance anti‐tumor immunity induced by the CMV pp65‐LAMP mRNA‐pulsed DC vaccine (NCT03688178).

Notably, all the DC‐based trials use autologous cells from patients' blood (Figure 4g), similar to the clinically approved DC product. In Provenge®, an FDA‐approved cancer vaccine for patients with metastatic castrate‐resistant prostate cancer, leukocytes are harvested from patients. These cells are expanded ex vivo in the presence of a prostate cancer tissue antigen and differentiated with GM‐CSF. Thereafter, the differentiated cell suspension, which mainly contains DCs, is injected intravenously into the patients.^85^ Such autologous DC vaccines have undergone comprehensive clinical investigation since the 1990s. This trend with DC vaccines for brain tumors is similar, as seen in the clinical trials for other tumor types.^86^ The time and resources needed to make such autologous DC vaccines remain a great challenge for translating these therapies.

### 3.3 Natural killer (NK) cells

**FIGURE 5:**
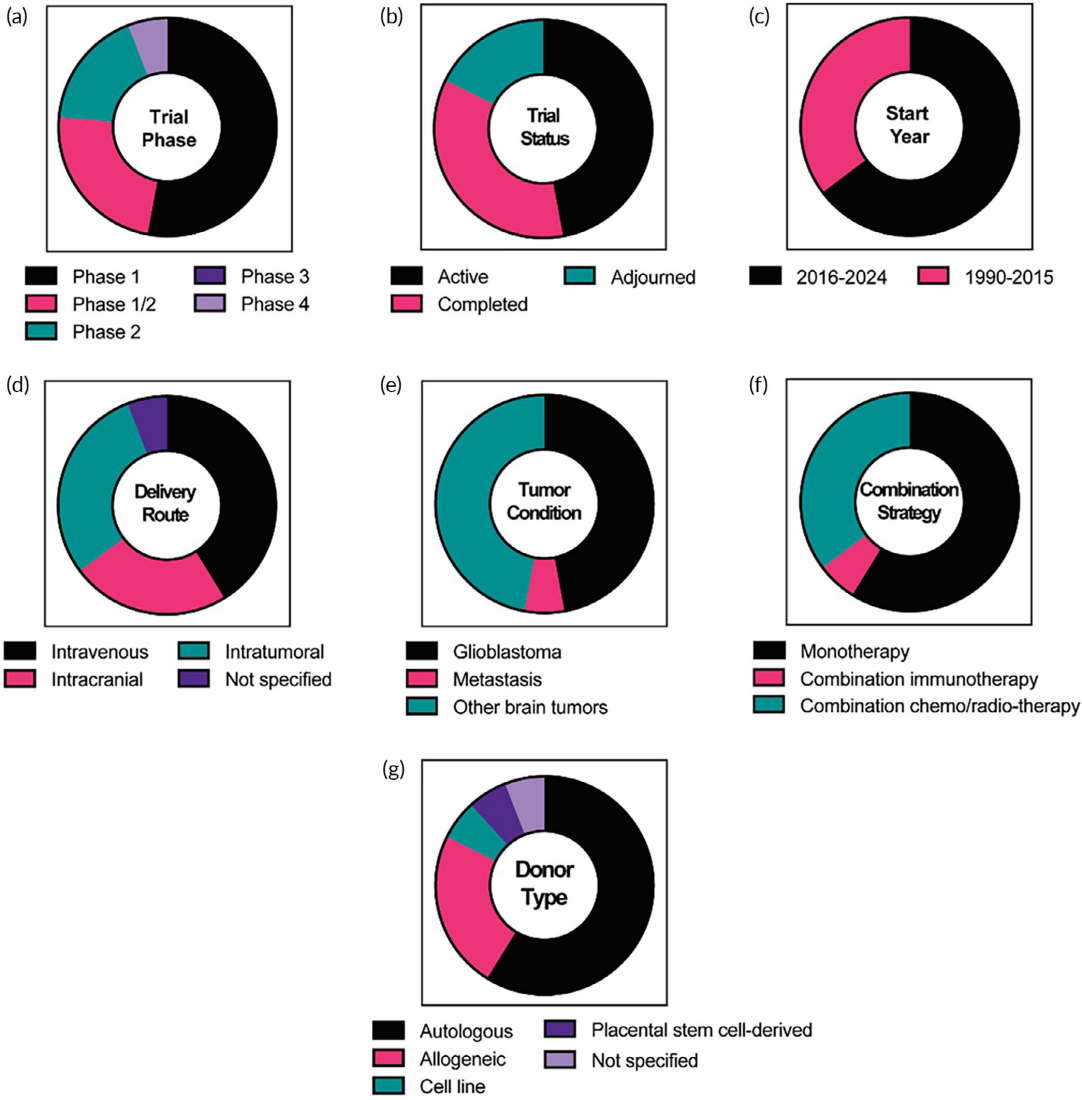
The current landscape of NK cell‐based clinical trials for brain tumor therapy. In total, 17 clinical trials were identified and analyzed according to (a) trial phase, (b) trial status, (c) start year, (d) delivery route, (e) tumor conditions (GBM, metastasis, or other types of brain tumors), (f) use of combination strategies, and (g) donor type.

Natural killer cells are the major innate lymphocytes that exert cytotoxic effects on tumor cells. They have important functions in anti‐tumor immune responses against diverse cancers, including brain tumors.^87^ Due to the distinct characteristics of NK cells, such as the major histocompatibility complex (MHC)‐independent anti‐tumor activity, the absence of GVHD, and the capability to prepare “off‐the‐shelf” therapeutic products, the use of NK cells has been gaining interest for brain tumor therapy as a promising alternate option to T cell‐based treatment. Intratumoral and intertumoral heterogeneities are one of the crucial challenges for T cell‐based GBM therapy.^52^ However, since NK cells recognize tumor cells without the need for recognition of specific tumor‐associated antigens, the employment of NK cells is a hopeful avenue for GBM treatment.^88^ At present, 8.4% of clinical trials for brain tumor therapy are occupied by NK cells (Figure 5), and some of those trials are presented in Table 3.

**TABLE 3:** Representative clinical trials using NK cells for treating brain tumors.

Start date (month/yy)	Status (Phase)	Clinical ID	Tumor condition	Cellular source	Injection route	Combined treatment
Apr‐2023	Not yet recruiting (Phase 1)	NCT04991870	Recurrent gliosarcoma, Recurrent Supratentorial GBM, Supratentorial Giosarcoma	Allogeneic (Cord blood‐derived expanded, TGFβR2 and NR3C1 depletion)	Intratumoral	Surgical resection
Dec‐2017	Not yet recruiting (Phase 1)	NCT03383978	Recurrent GBM (HER2‐positive)	NK‐92/5.28.z (CAR‐human NK cell line)	Intracranial	Ezebenlimab (aPD‐1), intravenous
Dec‐2023	Not yet recruiting (Phase 1)	NCT04254419	High‐grade glioma	Autologous	Intratumoral (Ommyama intra‐cavitary/intratumoral device)	NM
May‐2024	Recruiting (Phase 1)	NCT05887882	Pediatric brain tumor	Allogeneic (Universal donor TGFβi NK cell)	Intratumoral (with Ommyama reservoir placement)	
Jul‐2015	Not yet recruiting (Phase 1/2)	NCT02494804	Mallignant gliomas (Stage I‐II)	Autologous (CIK)	Intravenous	TMZ, oral
Jan‐2023	Recruiting (Phase 1/2)	NCT05588453	Brain metastasis	Allogeneic (Universal donor TGFβi NK cell)	Intravenous	TMZ, oral
Oct‐2023	Recruiting (Phase 1/2)	NCT06147505	GBM	NK cells (XS005)	Intracranial	TMZ
Jul‐2015	Not yet recruiting (Phase 4)	NCT02496988	Advanced malignant gliomas (Grade IV)	Autologous (CIK)	Intravenous	TMZ, oral
Mar‐2015	Completed (Phase 1)	NCT02271711	Recurrent medulloblastoma, Recurrent ependymoma	Autologous	Intraventricular	NM
Jun‐2013	Completed (Phase 1)	NCT01875601	Brain tumors, Neuroblastoma	Autologous	Intravenous	Recombinant human IL‐15, continuous infusion
Aug‐2011	Completed (Phase 1)	NCT01588769	GBM	Autologous	Intravenous	NM
Jan‐2009	Completed (Phase 1/2)	NCT00823524	Brain and CNS tumors	Autologous	Intravenous	NM
Feb‐2099	Completed (Phase 2)	NCT00331526	GBM	Autologous	Intracranial (Ommaya reservoir)	NM
Mar‐2013	Completed (Phase 2)	NCT02100891	Neuroblastoma, CNS tumors	Allogeneic	Intravenous	Allogeneic HSC transplantation

Abbreviation: NM, no mention.

Most trials for NK cells are in phase 1 (50%), which evaluates the safety and tolerability of the injected cells in order to determine a tolerable dose. Phase 1/2 (23.5%) and Phase 2 (17.6%) account for the rest of the trials, along with one phase 4 trial (Figure 5a). 47% of trials have active status (Figure 5b), with 65% of the trials started after 2015 (Figure 5c), suggesting an ongoing interest in investigating NK cells as future therapies for brain tumors. The injection routes hold immense importance for brain tumor treatment, with the unique challenge related to the BBB. The injection routes of the NK cell products in the trials are intravenous (41.2%), local delivery routes like intracranial/intraventricular (23.5%), and intratumoral (29.4%), with no scaffold‐based/depot‐based approach under clinical investigation (Figure 5d). For local delivery, four trials employ the Ommaya reservoir, which consists of a reservoir dome put on the brain surface under the scalp and a catheter providing access to the target region (NCT04254419, NCT00331526, NCT05887882, and NCT04489420).^89^ The device can be placed during therapeutic craniotomy or surgical resection procedures and enables long‐term treatment of brain tumors for repeated dosing. Similar to the trials using T cells, the proportion of trials employing locoregional infusion strategies for NK cells has dramatically increased after 2015, with 77.8% of the trials started after 2015 having been designed with intracranial or intratumoral administration.

The genetic engineering approach is emerging to augment the therapeutic benefit of NK cell products for a variety of cancer types, including brain tumors. In the phase 1 trial NCT03383978, CAR‐engineered NK‐92 cells are being used for patients with refractory or recurrent ErbB2 (HER2)‐positive GBM. A humanized CAR (CAR 5.28.z) targeting an ErbB2‐specific antibody with CD28 and CD3ζ signaling domains was transduced into the NK‐92 cell line.^90^ As increased ErbB2 protein expression is known in the brain tissues of GBM patients, this genetic modification allows the NK cell product to be specific and generate better therapeutic efficacy. This trial, which is the only trial utilizing the CAR‐NK cell therapy approach in GBM patients, was designed to establish the safety and feasibility of the approach following intracranial administration of CAR‐NK cells into the resection cavity during relapse surgery. The current trial protocol was amended after showing the safety and feasibility of intracranial administration of the CAR‐NK cells alone to investigate the combination of CAR‐NK92 cells with the anti‐PD‐1 antibody ezabenlimab.^91^ Notably, there is another phase 1 clinical trial employing genetically engineered NK cells (NCT04991870) but not CAR‐NK cells. This trial is examining the safety and feasibility of cord blood‐derived allogeneic NK cells as off‐the‐shelf products. The NK cells are genetically modified to contain deleted levels of transforming growth factor β receptor 2 (TGFβR2) and the glucocorticoid receptor (NR3C1). Depletion of TGFβR2 not only helps the NK cells to attack tumor cells but also to be resistant to the immunosuppressive cytokine TGF‐β in the cold TME of GBM.^92^ Depleting NR3C1 receptors from NK cells helps avoid dexamethasone treatment‐derived undesirable immunosuppressive effects. The administration of the systemic corticoid dexamethasone is widely used to treat cerebral edema and inflammation induced by brain tumors, but the treatment is found to be associated with immunosuppression, which can result in NK cell inactivity.^93^ Cytokine‐induced killer (CIK) cells, which possess both NK and T cell‐like phenotypes and MHC‐unrestricted anti‐tumor effector function,^94^ have also been used as another strategy. One of the distinct characteristics of CIK cells is that these cells are easily produced via ex vivo expansion of PBMCs by combined treatment with cytokines.^95^ CIK cells are utilized in the most progressed phase 4 trial for treating advanced malignant glioma (NCT02496988). The effectiveness and safety of CIK cells for stage I‐II malignant gliomas are also examined in a Phase 1/2 trial (NCT02494804).

NK cell trials are employed for all kinds of brain tumor types, with primary tumors, the majority of which are GBM, being highly favored compared to metastatic ones (Figure 5e). 62.5% of trials employ NK cells as monotherapy, while the majority of combination therapies are done with chemo/radiotherapy (Figure 5f). Combination with immunotherapy is still minimally tested as NK cells are in early development. It is expected in the future that NK cells will be tested in combination with cytokines and immune checkpoint inhibitors to see the synergistic therapeutic response. Unlike T cells and DCs, trials for NK cells employ a diverse variety of cellular sources, including autologous (57.1%), allogeneic (28.6%), and NK cell lines (7.1%) (Figure 5g). Since allogeneic NK cells can exhibit cytotoxic effects on tumor cells without affecting healthy cells,^96^ allogeneic NK cells offer a great opportunity to serve as an off‐the‐shelf product. For autologous and allogeneic NK cell trials, cells are typically derived from PBMCs, expanded, and activated ex vivo before treatment. However, the limited availability of NK cells from PBMCs (approximately 10% in peripheral leukocytes) and difficulty in both expansion and activation remain translational challenges to obtain sufficient numbers for treatment.^97^ NK cells derived from human placental CD34^+^ HSC were utilized to address these challenges in one trial (NCT04489420), although the trial was terminated. The limited expansion ability of the initial pool of primary cells while maintaining the stem‐cell‐like characteristics is mentioned to be one of the major challenges in using HSC‐derived NK cells. Another interesting approach in a phase I study is to use an established human cell line, NK‐92 cells (NCT03383978). The cells were derived from a patient with rapidly progressing non‐Hodgkin's lymphoma.^98^ As stem cell‐derived NK cell products and NK‐92 cell lines can extensively be applied for treating other cancer types, further development of stem cell‐ and NK cell line‐based technologies should enable overcoming translational challenges of NK cell‐based brain tumor therapy.

### 3.4 Stem cells

**FIGURE 6:**
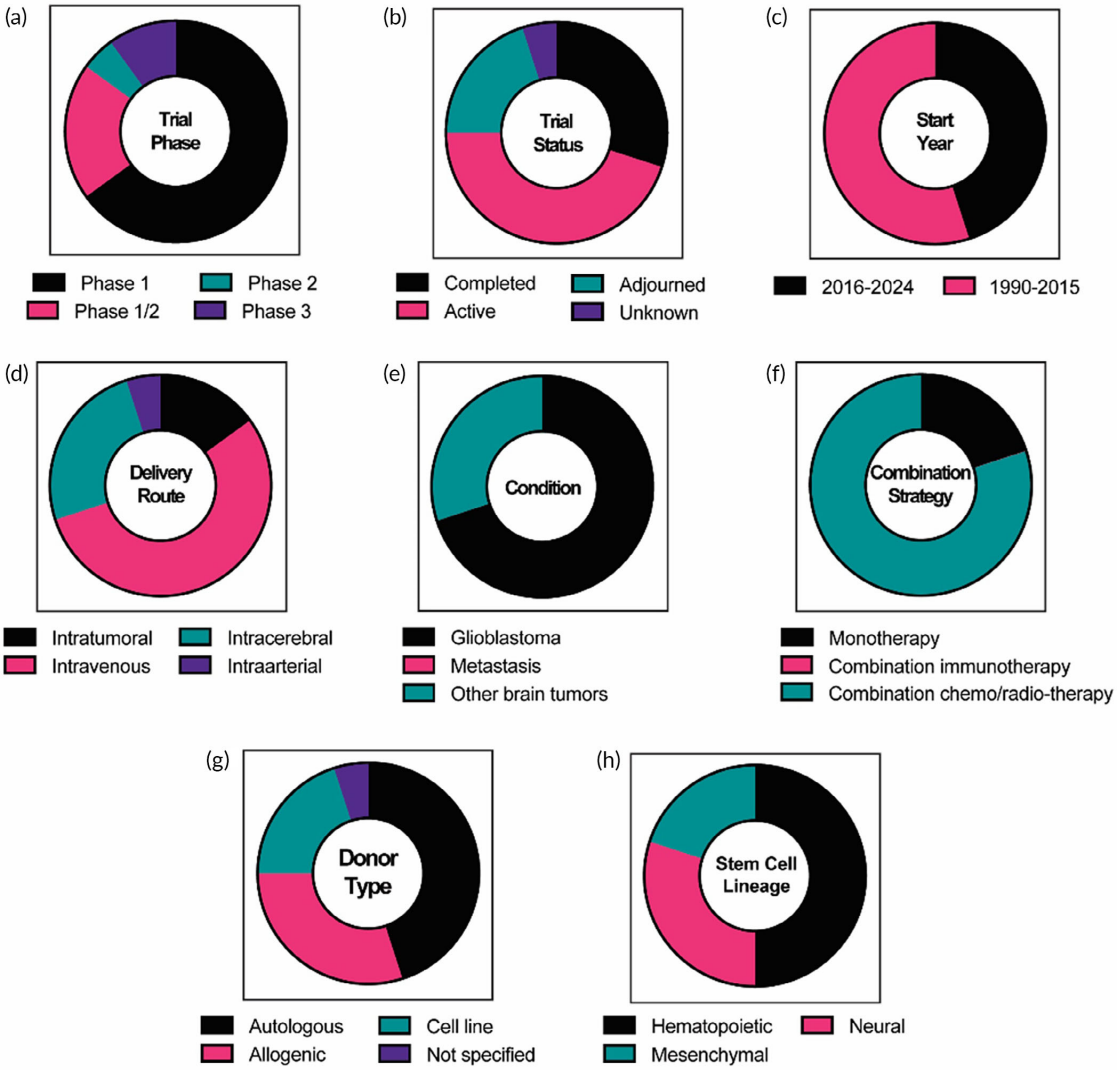
Landscape of stem cell‐based clinical trials. A total of 20 clinical trials (6 for NSCs, 10 for HSCs, and 4 for MSCs) were identified and analyzed regarding (a) trial phase, (b) trial status, (c) start year, (d) delivery route, (e) condition, (f) use of combination strategy, (g) donor type, and (h) stem cell lineages.

Stem cells are precursor cells that have the capability to differentiate into various cell/tissue types. They are categorized by their differentiation lineages. For therapeutic application to brain tumors, neural stem cells (NSCs), mesenchymal stem cells (MSCs), and HSCs have been employed. At present, 9.9% of clinical trials for brain tumor therapy are occupied by stem cells (Figure 6). They are summarized below in Table 4. As the action mechanism differs drastically between stem cell types, we will discuss them separately in this section.

**TABLE 4:** Examples of clinical trials employing stem cells for brain tumor therapy.

Start date (month/yy)	Status (phase)	Clinical ID	Tumor condition	Cellular source	Modification	Injection route	Combined treatment
*Neural stem cells (NSCs)*							
May‐2023	Recruiting (Phase 1)	NCT05139056	Recurrent GBM/gliosarcoma/malignant glioma, Recurrent anaplastic astrocytoma/oligoastrocytoma/oligodendroglioma	Not specified	Loading oncolytic adeno virus CRAd‐S‐pk7	Intracerebral	Surgical Resection
Mar‐2016	Active, not recruiting (Phase 1)	NCT02192359	Recurrent GBM/gliosarcoma/malignant glioma, Recurrent anaplastic astrocytoma/oligoastrocytoma/oligodendroglioma	Allogeneic	Genetic modification to express hCE1m6 (Adenoviral transduction of the hCE1m6)	Intracranial	Irinotecan (Intravenous)
Aug‐2010	Completed (Phase 1)	NCT01172964	Recurrent grade III‐IV glioma, GBM, Anaplastic astrocytoma/oligodendroglioma/oligoastrocytoma	HB1.F3 NSC cell line	Genetic modification (Transfection of *E.coli* CD gene)	Intracranial	Flucytosine (Oral)
Apr‐2017	Completed (Phase 1)	NCT03072134	GBM, Glioma, Anaplastic astrocytoma/oligodendroglioma/oligoastrocytoma, Astrocytoma (Grade III‐IV)	HB1.F3.CD21 NSC cell line	Loading oncolytic adeno virus CRAd‐S‐pk7	Intratumoral	Radiotherapy, temozolomide (Intravenous)
Oct‐2014	Completed (Phase 1)	NCT02015819	Recurrent GBM/gliosarcoma/high‐grade gliomas, Anaplastic astrocytoma/oligodendroglioma/oligoastrocytoma	HB1.F3 NSC cell line	Genetic modification (Transfection of *E.coli* CD gene)	Intracranial	Flucytosine (Oral), Leucovorin (Oral)
*Mesenchymal stem cells (MSCs)*							
Apr‐2021	Recruiting (Phase 1/2)	NCT04758533	Diffuse Intrinsic Pontine Glioma, Medulloblastoma (Childhood, Recurrent)	Bone marrow derived allogeneic	Loading oncolytic adenovirus ICOVIR‐5	Intravenous	
Feb‐2019	Recruiting (Phase 1)	NCT03896568	Recurrent Anaplastic astrocytoma/GBM/ Gliosarcoma/Malignant glioma	Bone marrow derived allogeneic	Loading oncolytic adenovirus Ad5‐DNX2401)	Intra‐arterial	
Jun‐2023	Not yet recruiting (Phase 1)	NCT05789394	Recurrent GBM	Adipose derived allogeneic		Intratumoral	Craniotomy
Jun‐2020	Completed (Phase 1/2)	NCT04657315	Recurrent GBM, Adult gliosarcoma	Not specified	Genetic modification (CD expression)	Intratumoral	5‐Flucytosine
*Hematopoietic stem cells (HSCs)*							
Aug‐2007	Active, not recruiting (Phase 3)	NCT00336024	Medulloblastoma	Autologous	NM	Intravenous	Carboplatin, Cisplatin, Cyclophosphamide, and others
Jan‐2023	Active, not recruiting (Phase 2)	NCT05052957	GBM, Supratentorial GBM, Supratentorial gliosarcoma	Autologous	Genetic modification (P140K‐MGMT)	Intravenous	O^6^BG, TMZ, Filgrastim, Camustine, Photon based radiotherapy
Mar‐2019	Recruiting (Phase 1/2)	NCT03866109	GBM	Autologous (Hematopoietic stem and progenitor cells)	Genetic modification (Myelloid specific IFN‐α2)	Intravenous	Thiotepa, Carmustine, Busulfan
May‐1997	Not yet recruiting (Phase 1)	NCT00007813	Neuroblastoma, Brain and CNS tumors	Autologous	NM	Intravenous	Cyclophosphamide, Filgrastim, Carboplatin, Etoposide
Nov‐2011	Completed (Phase 1)	NCT01269424	GBM	Autologous	Genetic modification (P140K‐MGMT)	Intravenous	O^6^BG, TMZ, Radiation therapy

Abbreviation: NM, no mention.

#### 3.4.1 Neural stem cells

Neural stem cells is a multipotent stem cell with the capability for self‐renewal and differentiation into neurons, oligodendrocytes, and astrocytes. Since NSCs have a high tropism for brain tumor cells with a remarkable ability to migrate deeply into tumor mass, they have been applied as delivery vehicles for several types of therapeutics, such as oncolytic viruses, enzymes, and cytokines, to treat brain cancer.^99^ The tumor‐targeting property of NSC was first demonstrated in 2000 using adult rodent brain tumor models,^100^ in which NSCs were injected locally into the brain. The NSC can invade and spread widely throughout the tumor. At present, we have found six clinical trials for NSCs to treat brain cancer, and those trials comprise 30% of all the trials using stem cells (Figure 6), as shown in Table 4.

All six trials utilizing NSCs were phase 1 studies (100%) to evaluate safety and determine the optimal dosage of NSC products for future phase 2 studies. Interestingly, none of the trials employed autologous cell sources. Two trials utilized allogeneic NSCs, while the rest of the four trials used a cell line HB1.F3 NSCs. The immortalized NSC cell line is obtained from the fatal telencephalon of pregnant women at 15 weeks of gestation.^101^ The HB1.F3 cells were generated by transducing the NSC cell line with *E. coli* to express cytosine deaminase (CD), which is the enzyme responsible for the conversion of the prodrug flucytosine (5‐fluorocytosine: 5‐FC) to the anti‐cancer agent 5‐fluorouracil (5‐FU). This cell line has received FDA approval to be used in clinical studies.^102^ All injection routes of NSC products are locoregional, with five trials administering cells intracranially, while one trial is carried out by intratumoral injection following surgical resection.

A notable feature of the clinical trials using NSCs is that all trials employ modified NSCs. A trial intracranially injecting genetically modified HB.1F3 NSCs expressing CD found no dose‐limiting toxicity, and injected NSC cells could migrate to distant tumor areas.^103^ In addition, the migrated cells converted an orally administered prodrug 5‐FC to 5‐FU selectively in intracerebral tumor tissues, indicating that the enzyme‐prodrug system adequately worked. This first‐in‐human NSC trial for patients with recurrent glioma provided proof of concept that NSCs modified genetically are relatively safe following local injection and distributed to target regions with desired functionality, although the trial could not indicate significant differences in median PFS and OS.^103^ In another trial utilizing HB1.F3.CD (NCT02015819), patients with recurrent high‐grade gliomas were intracranially injected with CD‐expressing NSCs in combination with flucytosine and leucovorin. Those combined drugs were given orally. A combination of leucovorin is expected to increase the therapeutic efficacy of 5‐FU converted from flucytosine. Another different type of the enzyme‐prodrug system is employed in the phase 1 trial (NCT02192359), in which allogeneic NSCs are genetically modified to express human liver carboxylesterase (hCE1m6) via adenoviral transduction.^104^ The NSC cell products are intracranially injected to high‐grade glioma patients together with intravenous infusion of the anti‐cancer drug irinotecan. The prodrug irinotecan is changed to SN‐38, a potent topoisomerase I inhibitor,^105^ at the brain tumor site by the presence of hCE1m6‐NSCs, which allows for more selective and effective treatment by co‐administered irinotecan. However, the BBB permeability of the irinotecan prodrug is low, which might be a challenge for achieving sufficient therapeutic response.

Another notable approach with modified NSCs uses NSCs loading an oncolytic adenovirus. In a phase 1 trial, CRAd‐Survivin‐pk7 (CRAd‐S‐pk7), a conditional replication adenovirus that brings about selectivity to brain cancer cells via the tumor‐specific survivin promoter and fiber modification,^106^ was loaded into the NSC cell line. In a first‐in‐human study utilizing these modified‐NSC cells (NCT03072134), both viral particles and NSCs were injected intratumorally into the walls of the cavity generated by neurosurgical resection in newly diagnosed malignant glioma patients, followed by initiation of the treatment with TMZ and radiotherapy within 10–14 days. This therapeutic regimen was found to be safe and feasible without treatment‐related deaths. These findings have helped in starting a phase 2 trial for investigating the therapeutic effectiveness of the approach.^107^ The safety and therapeutic response of multiple doses of this NSC product are also being examined for treating high‐grade recurrent gliomas (NCT05139056). In this trial, patients receive an intracerebral injection of NSC‐CRAd‐S‐pk7 after surgical resection once weekly, up to 4 doses without unacceptable toxicity or pathological progression. Compared with a single injection (NCT03072134), multiple doses of the NSC product are expected to kill tumor cells more effectively.

#### 3.4.2 Mesenchymal stem cells

MSCs are harvested from several tissues, including bone marrow, umbilical cord, adipose tissue, and placenta. Similar to the tumor tropism behavior of NSCs, many experimental studies have reported the brain tumor tropism and migration ability of MSCs in the orthotopic GBM models, independent of the injection routes.^108^ Importantly, there is evidence that systemically administered MSCs are able to pass through the BBB and infiltrate into brain tumor tissues.^109^ In addition, MSCs are massively expandable in vitro and possess immune‐evasive properties.^110^ MSCs are recognized as a promising source for cell‐mediated GBM therapy and are used to deliver diverse therapeutic modalities, including oncolytic viruses, suicide genes, and chemotherapeutic agents in many animal studies.^108^, ^111^ We found four clinical trials for MSCs for treating brain cancer, accounting for 20% of stem cell‐based trials (Figure 6 and Table 4).

Among the four trials, two trials are phase 1 studies (50%), and the remaining two trials are phase 1/2 (50%) for evaluating the safety and effectiveness of the MSC products. Although one trial does not specify the cellular source, the other three trials utilize allogeneic bone marrow‐derived (50%) or adipose‐derived MSCs (25%) as therapeutic cells. The tendency to utilize allogeneic cells reflects the trend of off‐the‐shelf manufacturing, which was seen both in other investigated MSC therapies for different diseases and recently approved MSC therapy (Alofisel) by the European Medicines Agency (EMA).^32^, ^112^ The injection route varies by trial; two trials employ intratumoral injection during surgical resection of recurrent GBM, while the remaining two are systemic injections.

Notably, three out of four trials (75%) utilize modified MSCs loaded with oncolytic viruses or the suicide gene. In an active phase 1 trial (NCT03896568), bone marrow‐derived allogeneic MSCs loading an oncolytic adenovirus Ad5‐DNX‐2401 (MSC‐DNX‐2401) have been used in recurrent high‐grade glioma patients. DNX‐2401 is modified to present arginine‐glycine‐aspartate (RGD) peptides, which bind to integrins with higher expression levels than natural adenovirus receptors on the glioma cell surface. This increases their tumor targetability and bioavailability while decreasing the risk of off‐target damage to the surrounding normal brain parenchyma.^113^ In addition, endovascular super‐selective intra‐arterial (ESIA) infusion is employed for the delivery of MSC‐DNX‐2401, which was demonstrated to be a superior approach to delivering therapeutic agents intracerebrally in comparison to the intravenous route.^114^ In another active phase 1/2 trial (NCT04758533), bone marrow‐derived allogeneic MSCs infected with ICOVIR‐5 (AloCELYVIR) have been used. The oncolytic virus ICOVIR‐5 is also modified to express the RGD motif to enhance its targeting of tumor cells. The safety of systemic administration of autologous bone marrow‐derived MSCs loading ICOVIR‐5 has been previously reported in a first‐in‐human trial.^115^ Genetically modified MSCs expressing a suicide gene CD (MSC11FCD) have also been utilized for recurrent GBM therapy in a phase 1/2 trial (NCT04657315). The MSC11FCD was intratumorally infused in the resection cavity during surgery with concomitant oral administration of a prodrug 5‐flucytosine, which is selectively converted to 5‐FU by CD‐expressing MSCs, similar to the studies using NSCs. It will be interesting to evaluate which of these two stem cell types with brain tumor tropism serves as a better choice for this prodrug‐converting technology in a head‐to‐head trial.

A phase 1 trial with non‐modified MSCs is performed utilizing allogeneic adipose‐derived MSCs (AMSCs) in treating patients with recurrent GBM (NCT05789394). Patients receive AMSCs intratumorally at the time of surgery. In preclinical studies, AMSCs have been demonstrated to exhibit both tumor tropism and anti‐tumor properties via several mechanisms, such as angiogenesis inhibition, apoptosis induction, and cell cycle modulation.^108^ Based on these advantages of AMSCs for GBM treatment, the trial expects that AMSCs will affect tumor growth, overcome resistance, and induce residual tumor cell death following local delivery into the surgical cavity, resulting in improvement of the long‐term outcomes of the patients.

#### 3.4.3 Hematopoietic stem cells

Hematopoietic stem cells are multipotent stem cells derived from mature blood cells of the myeloid and lymphoid cell lineages. HSCs can be obtained from mobilized peripheral blood, cord blood, and bone marrow. Other stem cell types are utilized for their anti‐tumor benefits against brain tumors, while HSCs, except for one trial, are mainly used as regenerative therapy to replace blood cells that are destroyed by previously administered chemotherapeutic drugs. Such rescue allows investigators to inject higher doses of chemotherapy to efficiently kill tumor cells. The process of HSC therapy consists of HSC collection, ex vivo purification, and cell engineering, followed by infusion into the patients to restore their hematopoietic function.^116^

There was a total of 10 clinical trials employing HSCs (50% of stem cell‐based trials). The majority of trials (9 out of 10) utilize autologous HSCs. 60% of trials use genetically modified HSC products. Among them, three trials use P140K‐methylguanine methyltransferase (MGMT) gene‐modified CD34+ HSCs to avoid hematopoietic toxicity induced by combination therapy with temozolomide (TMZ) and O^6^‐benzylguanine (O^6^BG) (NCT05052957, NCT01269424, NCT00669669). Almost 50% of GBM tumors possess resistance to TMZ due to MGMT overexpression (i.e., patients with unmethylated MGMT promoter).^117^ Coadministration of O^6^BG has been shown to effectively inhibit MGMT activity and restore the sensitivity of tumor cells against TMZ. However, serious off‐target myelosuppression resulting from low to absent levels of MGMT in HSCs and progenitor cells is observed in the combination of TMZ and O^6^BG. Expression of P140K‐MGMT mutant renders HSCs resistant to O^6^BG treatment, resulting in significant protection against hematopoietic toxicity by combination with TMZ and O^6^BG.^118^

One trial utilizes autologous HSCs modified genetically with a specific lentiviral vector encoding for the human interferon (IFN)‐α2 gene (NCT03866109). The gene expression is regulated by the human TIE2 enhancer/promoter sequence and a post‐transcriptional regulation layer represented by sequences of target miRNA. This allows for the expression of IFN‐α2 only in the TME of GBM tissue via Tie2‐expressing myeloid cell progeny monocytes generated from transferred HSC.^119^ Immunomodulation of the TME in GBM tissues by delivered IFN‐α2 is expected to increase the therapeutic benefits of second‐line therapies such as surgery and anti‐cancer chemotherapies.

### 3.5 Monocyte

Monocytes are one of the myeloid immune cells and the precursors of macrophages. Monocytes are known to cross the inflamed endothelial barrier, including the BBB, reach hypoxic regions of tumors, and finally differentiate into tumor‐associated macrophages (TAM) in the tumor tissues.^120^ By focusing on these properties, the application of monocytes for anti‐cancer drug delivery to brain tumor regions has been reported for treating GBM‐bearing mice,^121^ although there is no report on utilizing monocytes themselves as therapeutic cells for GBM treatment. Preclinical studies utilizing monocytes or macrophages for inflammatory and degenerative diseases have been reported.^122^ However, their translation to the clinic has been limited to date.

We could find one completed phase 1 trial utilizing lymphokine‐activated monocytes combined with bispecific antibodies for treating recurrent or refractory GBM patients (NCT00005813, completed, start date: Mar‐97). The trial performed combination treatment with bispecific antibody MDX447 and activated monocytes intratumorally within 2–4 weeks after conventional surgery. MDX447 is a bispecific antibody targeting an Fc receptor (FcγRI/CD64) and epidermal growth factor receptor (EGFR). Fc receptors are expressed on immune cells, including monocytes, while EGFR is overexpressed in tumors of most primary GBM patients.^123^ The preclinical studies demonstrated that MDX447 simultaneously binds to both FcγRI and EGFR and then induces antibody‐dependent cell‐mediated cytotoxicity (ADCC) to kill GBM cells overexpressing EGFR.^124^ As monocytes can migrate into tumor tissues, both MDX447 and activated monocytes were expected to locate around tumor cells and kill them via ADCC after intratumoral injection. Another phase 1 study used monocytes loaded with CMP pp65‐LAMP mRNA as vaccines to determine the safety; however, the trial was withdrawn due to the need to improve the technical and economic feasibility (NCT04741984, withdrawn, start date: Aug‐23). It is surprising to see that the immune cell type, which is so predominantly present in brain tumor tissues, is leveraged very minimally for brain tumor treatment. Monocytes differentiate into macrophages upon infiltrating tissue sites. However, the immunosuppressive TME skews this polarization towards an anti‐inflammatory phenotype, which promotes pro‐tumorigenic effects.^125^ While adoptive cell therapies using macrophages with the anti‐tumor phenotype have been explored for treating renal, ovarian, colorectal, and non‐small‐cell lung cancers, a favorable clinical outcome was not achieved with these treatments.^126^ One of the key challenges limiting the therapeutic efficacy of macrophage‐based therapies is their transport limitation within the tumor, alongside their dynamic phenotypic plasticity between anti‐inflammatory and pro‐inflammatory states.^127^, ^128^ A recent study demonstrated that monocytes exhibit superior trafficking to immunosuppressive tumors following intravenous administration compared to macrophages.^129^ These findings suggest that developing technologies to maintain the anti‐tumor phenotype of tumor‐infiltrating monocytes within the immunosuppressive TME could promote their clinical translation. Advancing such technologies may facilitate the successful translation of monocyte‐based therapies for solid tumors, including GBM.

## 4 LEARNING FROM CLINICAL TRIALS AND APPROACHES TO ADDRESS CLINICAL CHALLENGES

According to our analysis discussed before, over 50% of all trials are still phase 1 studies, whereas several trials have progressed to phase 2 or later. Notably, a few trials utilizing T cells (NCT05685004, NCT00807027) and DC (NCT05100641, NCT00045968, NCT03548571) have reached phase 2/3 or 3, and the most advanced NK cell trial (NCT02496988) is currently in phase 4. These trends indicate the safety and feasibility of some of the approaches utilizing therapeutic cells and suggest that cell therapy could be a promising avenue to induce effective therapeutic responses for treating brain tumors, especially GBM. However, several challenges have emerged from the clinical experience of using cells as a therapy for brain tumors. These challenges can be broadly categorized into 1) selection of target antigens, 2) viable route of administration, 3) persistence of administered therapeutic cells, and 4) safety of allogeneic “off‐the‐shelf” cell products, among others. We discuss these challenges and highlight promising approaches to overcome them in this section.

### 4.1 Selection of targeting antigens

Approaches targeting tumor‐associated specific antigens have been employed in a lot of clinical trials utilizing CAR‐expressing T/NK cells and DC‐based cancer vaccines. With high inter‐patient and intra‐tumor heterogeneity observed in GBM tissues, the selection of the right target antigen has paramount importance. Since CARs only recognize cell surface proteins, elucidation of proper and specific antigens is an essential constraint for CAR‐engineered cell therapies. In clinical trials with CAR‐T cell therapies, EGFRvIII, HER2, and IL‐13Rα2 have often been targeted. However, EGFRvIII is known to be expressed in only approximately 30% of the patients, and its expression across different patients is heterogeneous.^130^ HER2 targeting is also employed in various clinical trials. HER2 is overexpressed more frequently and homogenously compared to EGFRvIII. However, the expression of HER2 in healthy epithelial cells limits the therapeutic window because of the possibility of on‐target off‐tumor adverse effects.^53^ IL‐13Rα2 is expressed frequently in over 50% of GBM, and its overexpression correlates with poor prognosis.^131^ Transient improvement of patient outcomes has been reported with CAR‐T cells targeting IL‐13Rα2,^59^ although the overall clinical benefit has not been clear. In addition to these targets, several recent phase 1 trials have been utilizing CAR‐T cells targeting new antigens, including B7H3, MMP2, NKG2DL, and CD70. An immune checkpoint molecule, B7H3, is overexpressed in GBM tissues with minimal expression in normal tissues.^132^ B7H3 is one of the hopeful targets since the protein is expressed highly and widely in tumor tissues of patients with GBM.^132^ Membrane‐associated MMP2 is another broadly expressed antigen in human GBM tissues.^133^ A 36‐amino acid venom‐derived peptide, Chlorotoxin (CLTX) possesses the capability to bind MMP2 on GBM cells.^134^ Although CLTX itself does not exhibit cytotoxicity to tumors, the utility of CLTX as a safe glioma‐specific targeting tool has been demonstrated in clinical trials.^135^ CAR‐T cells displaying CTLX showed high anti‐tumor activity via strong binding to GBM cells and also to GBM stem cells (GSCs) in a preclinical study.^134^ Since GSCs have important roles in maintaining the aggressive phenotype of GBM and are considered highly potential targets, the results of the ongoing phase 1 study with this approach will be of great interest. NKG2DL is another marker highly expressed in both GBM tissue and GSCs. CAR‐T cells engineered with the extracellular domain of NKG2D are expected to become a hopeful cell therapy against GBM.^136^ Additionally, tumor antigens or mRNA derived from autologous GSCs are also employed to target GSCs in ongoing trials involving DC vaccines (NCT03548571 and NCT4888611).

Targeting multiple antigens is emerging to be a hopeful strategy for addressing challenges related to tumor heterogeneity of GBM since identifying specific and homogenously expressed antigens has been extremely difficult. This approach is mainly investigated in T‐cell and DC‐based therapy. Regarding CAR‐T cell therapy, only a few phase 1 trials have utilized CAR‐T cells that target multiple antigens (NCT05577091, NCT05868083, and NCT05168423). One example is Tris‐CAR‐T cells (NCT05577091), which are designed for targeting both CD44 and CD133 and include an intracellular domain with truncated IL7Rα. Another cell product, termed SNC‐109, is genetically engineered to express CARs targeting HER2, IL13Rα2, and EGFRvIII (NCT05868083). Additionally, another T cell product that co‐expresses two CARs targeting the cryptic EGFR epitope 806 and IL13Rα2 is also being investigated (NCT05168423). Although brain tumor cells can evade immune recognition via antigen escape mechanisms such as selective survival of antigen‐negative subpopulations, deletion/down‐regulation of targeted antigens, and antigen mutation, multi‐specific CARs can minimize antigen escape and enhance T‐cell effector function, offering potential for more effective GBM therapy.^62^ On the other hand, the multiple antigen targeting strategy needs to address several associated challenges such as increased toxicity from immune‐related adverse events, faster immune exhaustion, and the complexity of engineering processes. To enhance safety, strategies such as incorporating an inducible caspase‐9 suicide gene already utilized in clinical trials for certain solid tumors, including brain tumors (NCT03373097, NCT02414269, and NCT04196413), or employing reversible on/off switch CARs could provide initial safety improvements.^137^, ^138^ Another approach is the SynNotch system, which allows CAR expression only upon recognition of combinatorial tumor antigens. This system is currently being evaluated in a Phase 1 trial for GBM (NCT06186401). To mitigate CAR T cell exhaustion within the TME, direct targeting of the immunosuppressive nature of the TME has been explored. In particular, the adoptive transfer of TGF‐β‐resistant CAR T cells targeting prostate‐specific membrane antigen in patients with metastatic castrate‐resistant prostate cancer (mCRPC) resulted in a >30% reduction in prostate‐specific antigen levels in four of 13 patients.^139^ Additionally, switch CAR T cells, which convert inhibitory signals to T‐cell activation, are also being studied for mCRPC therapy (NCT06046040). Engineering CAR T cells to secrete PD‐1‐specific antibodies or nanobodies upon activation has also been investigated for patients with solid tumors (NCT05373147 and NCT05089266).^140^ While CAR T cells targeting multiple antigens hold significant promise for treating cancers with heterogeneity, the complexity of their engineering processes is a challenge. Clinical and regulatory efforts are underway to develop faster, distributed, and automated manufacturing to reduce costs and improve efficiency.^141^, ^142^ Moreover, intensive efforts to directly engineer CAR T cells in vivo as well as to use engineered off‐the‐shelf cell products may streamline the complex manufacturing processes and increase the accessibility of CAR T‐cell therapy products.^143^, ^144^

Regarding DC‐based cancer vaccines, a lot of active clinical trials are exploring multi‐targeting approaches using autologous tumor antigens, total tumor RNA (TTRNA), and multiple tumor antigen peptides (NCT04968366). Using whole tumor antigens is particularly advantageous, since they contain a diverse array of patient‐specific tumor antigens such as protein antigens, lipids, and carbohydrates, which allows for a more comprehensive anti‐tumor immune responses in comparison to other approaches. In addition, targeting whole tumor antigens reduces the likelihood of immune evasion, even in the presence of tumor heterogeneity. Further, this approach also enables DCs to display post‐translationally modified, non‐mutated tumor antigens, which are difficult to predict using conventional vaccine approaches.^145^ A major clinical challenge with whole tumor‐derived antigens is the difficulty in the purification, preparation, and quantification of the DC product. As tumor lysates contain both immunogenic and non‐immunogenic antigens, developing methods to ensure the efficacy and safety of each DC vaccine while standardizing the manufacturing across patients is essential.

### 4.2 Viable Route of administration

The administration route of cell products varies depending on the cell types, with trials employing either intravenous or locoregional infusion approaches. To date, there is no approach for implanting a scaffold or depot loaded with cells for brain tumor treatment in clinical trials. Intravenous injection remains the most accessible method for injection. Intravenously injected immune cells have a better capability to enter the CNS by responding to biological cues compared to non‐living therapeutics. However, systemic infusion into the bloodstream faces significant challenges, such as inefficient migration across the BBB and blood‐CSF‐barrier, and limited infiltration through the dense tumor stroma, making this approach less effective for cell therapies targeting brain tumors.^146^ Localized delivery of cells can bypass the BBB and thus maximize therapeutic benefits by considerably increasing effector‐to‐target ratios in the tumor tissue. As a result, many recent active trials involving T cells, NK cells, and stem cells increasingly favor local delivery approaches. The locoregional treatment provides a unique opportunity for brain tumors as 90% of cases are diagnosed at the locoregional stage, with extracranial metastasis occurring in only 0.4%–2% of cases.^147^ Based on the accumulating data from clinical trials, especially using T cells, the transfer of therapeutic cells into the brain can be achieved with tolerable safety, even in the case of their direct injection into the brain. In addition, albeit only in one reported case, multiple intraventricular injections of CAR‐T cells targeting IL‐13Rα2 completely regressed recurrent multifocal GBM,^59^ indicating the importance of considering this administration route. The various modes of locoregional administration into brain tumors include intratumoral infusion into the tumor/resection cavity and intraventricular administrations to deliver cells into CSF via the lateral ventricle. These administration methods include needle‐syringe‐based direct injections or implanted catheter port/reservoir device systems. Strategies for implanting reservoir/catheter delivery devices can allow for multiple administrations. Implantation of the Ommaya reservoir (NCT05459441, NCT04077866) during resection surgery of brain tumors has been a widely used strategy in clinical trials.

DC vaccines utilize intradermal injection as a preferred administration route in clinical trials. However, one limitation of this route seen in melanoma patients is that intradermally administered DCs exhibit limited migration, with <5% of the injected cells reaching the drainage lymph nodes.^148^ To improve DC migration into cervical lymph nodes (CLNs) in patients with GBM, in some trials, intradermal injection was performed at a place close to CLNs (NCT04888611, NCT04388033) or direct intranodal injection is used (NCT00323115). The data demonstrating the superiority of those approaches have not yet been published. In another approach, vaccine site preconditioning using Td toxoid was demonstrated to significantly increase tumor antigen‐specific C migration to lymph nodes.^149^ Since deep CLNs are crucial for priming and activating antigen‐specific T‐cells and subsequently inducing a robust anti‐tumor immunity in meningeal lymphatics,^75^ the above‐mentioned approaches present promising avenues for addressing the clinical limitations of DC vaccines.

### 4.3 Persistence of administered therapeutic cells

The strongly immunosuppressive TME induces exhaustion of tumor‐infiltrating T and NK cells, resulting in a drastic reduction in their effector function, continuous inhibitory receptor expression, and lower production of effector cytokines (e.g., IFN‐γ, TNF‐α, IL‐2, and granzyme B).^150^ Tumor‐infiltrating T cells in patients with GBM express greater amounts of exhaustion‐related proteins including CTLA‐4, PD‐1, and TIM‐3, compared to blood‐circulating peripheral T cells and exhibit poor functional potential.^151^ Inhibitory cell‐surface antigens, including PD‐1/PD‐L1, and immunosuppressive cytokines (TGF‐β, IL‐10, and prostaglandin E2) present in brain tumors also suppress the activity of administered cells.^152^ Hence, even if adoptively transferred T‐cells reach the GBM tissue, the immunosuppressive environment induces T‐cell dysfunction and shows resistance to the treatment. The use of combination therapy with immune checkpoint inhibitors holds great promise for making adoptively transferred T‐cells resistant against immunosuppression and exhaustion. Some clinical trials have been carried out utilizing combinations with CAR‐T cells and checkpoint inhibitors (NCT04003649, NCT03726515). As immune checkpoint inhibitors decrease negative regulatory pathway activity which limits the activity of T cells, their combination with T‐cell therapy has the potential to increase the therapeutic outcome for patients with GBM. The combination strategy is also a hopeful avenue for avoiding NK cell dysfunction by the immunosuppressive TME and maintaining tumor‐killing activity of the cells. Combination with an anti‐PD‐1 checkpoint inhibitor is being investigated in an active phase 1 trial utilizing CAR‐NK cells to invigorate anti‐tumor immune responses (NCT04254419). Previous preclinical studies have demonstrated that anti‐PD‐1 inhibition enhanced immune responses caused by HER2‐targeting CAR NK cells, resulting in successful treatment of advanced GBM refractory to monotherapy.^153^ For DC vaccine therapy, making non‐exhausted and sufficient T cells reach the TME is a crucial challenge to treat primary brain tumors. For this purpose, combining immune checkpoint inhibitors is currently under investigation to augment the effect of DC vaccines (NCT03879512, NCT04201873, NCT04348747, NCT04888611, NCT0545795, and NCT02529072). Although there are no published data on the trials employing combined treatment with DC vaccines and immune checkpoint inhibitors, the results in preclinical models and the preliminary data in early state trials were mentioned to be promising in the trial NCT03879512.

Another way that has been employed to avoid exhaustion of the therapeutic cells is a gene‐editing approach to knock out genes involved in T cell exhaustion. In one preclinical study utilizing the CRISPR‐Cas9 system, CAR T cells having resistance to checkpoint signaling were created by knocking out PD‐1, endogenous T‐cell receptor, and beta‐2 microglobulin. The intracranial infusion of the gene‐edited CAR T cells targeting EGFRVIII, but not intravenous infusion, prolonged survival of the GBM model mice compared with non‐edited CAR T cells.^154^ In one case from a chronic lymphocytic leukemia patient, not a brain tumor patient, it was reported that CAR T cells targeting CD19 with induced translocation methylcytosine dioxygenase 2 (TET2) dysfunction could induce effective anti‐tumor responses. TET2 dysfunction produced strong CAR T cells with characteristics of both short‐lived effector memory T cells and long‐lived central memory ones.^155^ This finding also indicates an advantage of a strategic gene‐editing approach for improving the effectiveness of adoptive cell therapies. The result of the first‐in‐human phase 1 trial of CRISPR‐Cas9‐mediated multiple gene editing for engineering T cells was recently reported in three patients with refractory non‐brain cancers (NCT03399448).^156^ In this trial, two genes encoding the endogenous TCR chains (TCRα and TCRβ) and the PD‐1 gene were deleted in autologous T cells to reduce TCR mismatches and to improve antitumor immunity, respectively. Additionally, a synthetic TCR was transduced for targeting NY‐ESO‐1 (cancer testis antigen). When the modified cells were re‐infused into the patients, they safely persisted for up to 9 months and resulted in tumor evasion. Although the target cancer was not GBM in this trial, a similar gene‐editing approach may apply to GBM patients' derived T cells.

Gene editing is also employed for NK cells to avoid their dysfunction. Although a currently active phase 4 trial using cytokine‐induced killer (CIK) cells (NCT02496988) is promising, the tumor‐killing function of CIK cells is suppressed by brain tumor cells through the expression of certain factors including TGF‐β and PD‐L1, which contribute to forming the immunosuppressive TME.^157^ Especially, TGF‐β impairs the function of NK cells through downregulation of NKG2D and NKp30 (activating NK receptors).^158^ To circumvent the TGF‐β‐mediated exhaustion of NK cells, cord blood‐derived NK cells that express the dominant‐negative form of the TGF‐β receptor were generated and found to be therapeutically better at killing GBM cells.^92^ The phase 1 trial employing these genetically engineered NK cells is ongoing for treating recurrent GBM (NCT04991870). It will be interesting to see whether this NK cell product shows promising therapeutic outcomes in the clinics. The approaches for genetic blockage or deletion of checkpoint molecules for NK cells, such as IL‐1R8 or cytokine‐inducible Src‐homology‐2 containing protein (CIS), have also been performed for the treatment of several cancers but not for brain tumors.^159^, ^160^ Future studies are expected to apply these gene editing techniques to brain tumor therapy.

### 4.4 Safety of allogeneic off‐the‐shelf products

Achieving off‐the‐shelf cellular products for brain tumors is key to overcoming the logistical challenges of deploying such therapies. Currently, all DC trials use autologous cells, similar to the clinically approved products, Provenge, CreaVax, and APCeden, although these products are not indicated for brain tumors. Patient's autologous cells are used in most T‐cell trials. However, such treatments with autologous cells have notable limitations. Since the cell products need to be generated using cells from each patient, the process is time‐consuming and resource‐intensive which poses the risk of manufacturing failures, especially in cases of multiple infusions. The difficulty in establishing standardized preparation methods also creates issues for regulatory approval of the product.^161^ Further, in instances where patients have already received lymphodepleting chemotherapy and/or radiotherapy, they often have T cell dysfunction, resulting in poor quantity and quality of the starting T cells.^162^ The use of allogenic cell sources is another avenue that is pursued to resolve this issue. The use of healthy donor‐derived allogeneic T cells is expected to allow for obtaining higher amounts of functional cells and generating “off‐the‐shelf” CAR T cell products.^163^ However, treatment with allogeneic CAR T cells has the risk of inducing GVHD because of immunogenicity and alloreactivity, which could also impede their anti‐tumor efficacy. Some efforts have been made to avoid such GVHD and immune rejection to develop allogeneic CAR T cell therapies. One approach is to genetically modify cells for eliminating HLA‐I/‐II molecules and TCR by using CRISPR/Cas9 or other methodologies, such as TALENs and megaTAL nucleases.^164^ For instance, CRISPR/Cas9 was applied to produce HLA‐I and TCR deficient allogeneic CAR T cells with additional knockout of PD‐1. This brought about the resistance to immunosuppression while reducing CAR T cell alloreactivity.^165^ Another way to generate allogeneic CAR T cells is using a poorly alloreactive source or subset of T cells. One candidate is the use of γδT cells (only 1%–5% of circulating T cells), which are expandable ex vivo. γδT cells recognize target cells independently of MHC restriction, infiltrate into the tissue of solid tumors, and show potent cytotoxic activity.^166^ Indeed, phase 1 and 2 clinical trials utilizing γδT cells are ongoing in GBM patients (NCT05664243, NCT04165941). Preclinical studies with CAR γδT cells have shown promising results,^167^ which may soon enter clinical trials. Another possible source of allogeneic T cells is umbilical cord blood (UCB). T cells derived from HSCs enriched from UCB lack surface expression of TCRαβ complex, resulting in immune resistance and low GVHD incidence.^168^ Also, HSCs can differentiate into T cells at a higher ratio than manufacturing from peripheral blood‐derived autologous T cells.^169^ A high percentage of γδT was reported to be isolated from UCB, possibly because of the lack of thymic cortical epithelial cells involved in positive TCRαβ selection. Another study showed that UCB‐derived CAR T cells possessed more naïve T cells and lower Treg population. Such CAR T cells exhibited longer tumor suppression than patient's peripheral blood‐derived CAR T cells.^170^ Induced pluripotent stem cells (iPSCs) are expected to be another potential source of allogeneic T cells because iPSCs have infinite capability to self‐renew and clonally expand, which may bring about a completely homogeneous cell product.^171^ Although the use of iPSCs has several challenges, such as unexpected genetic modifications and safety concerns,^172^ universal CAR T cell therapies can be realized if the gene‐editing process is optimized. The establishment of allogeneic CAR T cells is quite a major challenge for treating all types of cancers, not limited to brain tumors, and more detailed content has also been summarized in other review papers.^173^

In contrast to T cell and DC approaches, allogeneic cell sources have been employed more prevalently in clinical trials using NK cells, creating a higher possibility of having off‐the‐shelf products. Since allogeneic NK cells derived from PBMCs of healthy donors possess greater anti‐tumor activity than exhausted NK cells derived from immune‐suppressed GBM patients, the use of allogeneic cell sources is considered advantageous in NK‐based GBM therapy.^174^ Although the trial using human placental HSC‐derived NK cells was terminated due to a business decision (NCT04489420), the trials using cord blood‐derived (NCT04991870) and universal donor‐derived (NCT05588453) NK cells are still active. Also, NK92 cells modified with CAR editing have been investigated in an ongoing trial (NCT03383978). Importantly, NK cells do not cause GVHD because they lack TCRs, while GVHD is a challenging issue in allogeneic T cell therapy, as mentioned above.^175^ Additionally, CAR NK cells are reported to be safer than CAR T cells due to the lower probability of cytokine release syndrome (CRS) and neurotoxicity.^175^ Considering these findings, NK cells are an ideal allogeneic cellular source to realize “off‐the‐shelf” products for brain tumor therapy. Nevertheless, the advancement of allogeneic NK cell therapies is hampered by some limitations. Since the NK cell proportion in circulating lymphocytes in adult humans is only 5%–15%, the difficulty of ex vivo expansion to yield enough cells remains a challenge. Transfecting NK cells with the CAR constructs to prepare CAR NK cells is reportedly more difficult compared with that of T cells, while gene editing with CRISPR Cas9 technology can overcome the problems related to transfection efficiency.^176^ In addition, automation and scaling up of the manufacturing processes are required to promote off‐the‐shelf product development.

## 5 SELECTED PRECLINICAL STUDIES, TECHNOLOGIES, AND OUTLOOK

**FIGURE 7:**
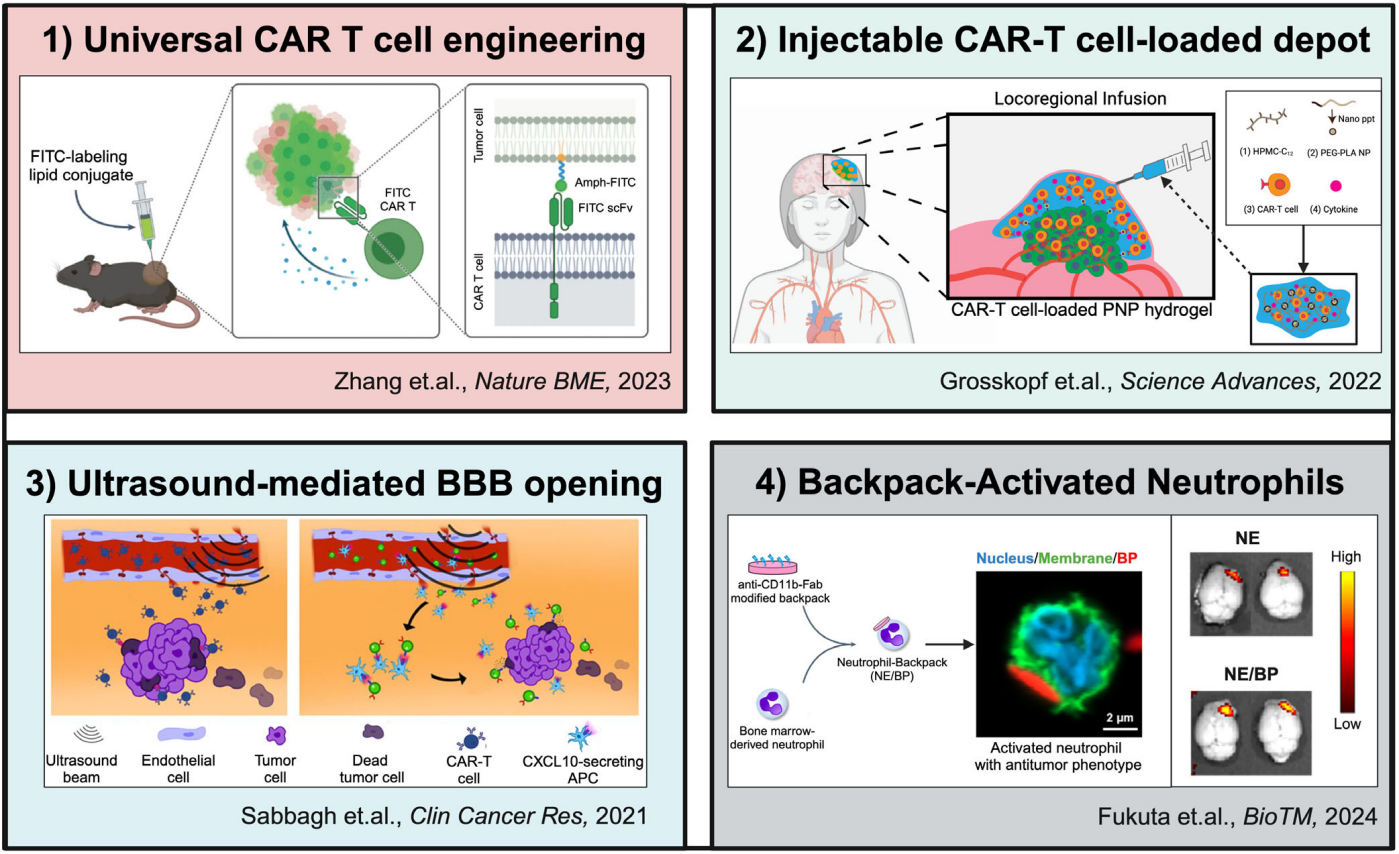
Schematic summaries of selected promising preclinical studies. (1) Universal CAR T cell engineering approach to develop a targeted cell therapy independent of endogenous antigen expression. Adapted with permission from Ren et al.^165^ (2) Locoregional administration of injectable CAR‐T cell‐loaded depot to ensure long‐term cellular persistence and functionality. Adapted with permission from Capsomidis et al.^167^ (3) Ultrasound‐mediated BBB opening to increase delivery efficiency of systemically injected therapeutic cells. Adapted with permission from Liu et al.^170^ (4) Backpack‐activated neutrophils, a material‐based cell‐surface engineering approach to induce systemic antitumor immune responses. Adapted with permission fromLiu et al.^175^

New approaches in the preclinical setting are emerging with the potential to address the challenges for clinical translation of brain tumor‐targeting cell therapies. We discuss some examples of these recent preclinical studies in this section. Schematic summaries of this section are presented in Figure 7.

### 5.1 Genetic modification approaches

Genetic engineering has been a primary strategy for cell modification to augment the anti‐tumor functions of therapeutic cells. Numerous innovative genetic engineering strategies have emerged in recent years to overcome challenges of cell therapies for brain tumors. One strategy involves designing CAR gene constructs with downstream supporting molecules such as cytokines, checkpoint inhibitors, and chemokines.^177^, ^178^ Given that GBM tumors are highly heterogeneous, another promising approach has been genetically modifying immune cells, particularly T cells, to target tumor‐associated multiple antigens simultaneously. Bi‐specific, tri‐specific, and even quad‐specific targeting strategies have been reported to address the heterogeneous brain tumor microenvironment.^179^ Additionally, genetic modification for targeting new antigen targets such as CAIX, CD70, CSPG4, and TROP2 is also being explored in preclinical studies.^52^ Beyond T cells, innate immune cells including NK cells and macrophages are being genetically engineered with CAR constructs to exploit their unique immunological and manufacturing advantages over adaptive T cells.^157^, ^180^ One innovative approach involves generating a universal CAR T cell that can target any tumor without intolerable toxicity for normal tissues. This strategy involves labeling tumor cells with a foreign marker, which is then targeted by CAR T cells against the foreign marker.^181^ A challenge in this strategy has been to deliver the labeling moiety specifically and sufficiently to label only cells of interest. A recent study revealed its potential using fluorescein isothiocyanate (FITC) to label solid tumor cells via intratumoral administration, followed by the injection of FITC‐targeting CAR T cells.^182^ In multiple syngeneic and human tumor xenograft models, this method showed a robust effect not only against primary labeled tumors but also against distal unlabeled tumors, as well as providing protection against tumor rechallenge. This transformative approach may offer treatment options that are independent of antigen expression and tumor location, an especially critical consideration for brain tumor treatments.

### 5.2 Administration method of therapeutic cells

Clinical experience with cell therapies for brain tumors has shown that the delivery route is crucial to their success.^35^ Systemic delivery faces significant challenges in achieving adequate infiltration and homing of transferred cells to the brain. Thus, a locoregional infusion is becoming a preferred approach in clinical trials for brain tumor treatment. However, ensuring long‐term cellular persistence and functionality within the immunosuppressive microenvironment remains a challenge for locally injected cells. The locoregional depot‐based strategies using biomaterial scaffolds to deliver cells along with supporting biomolecules are being explored to mitigate these issues, although they are still in the preclinical stage. Recent work from Ogunnaike et al.^183^ developed a CAR‐T cell‐loaded fibrin‐based injectable gel to maintain cell viability and achieve gradual release of cells in the tumor cavity. In their studies with the GBM mouse model, the researchers revealed that delivering CAR‐T cells with the fibrin gel achieved better efficacy than CAR‐T cells alone in inhibiting the growth of tumors. Notably, 64% of mice treated with the depot‐based delivery strategy were tumor‐free, compared to 20% tumor‐free survival in mice treated by free cell injections. In this study, no additional supporting factor in the depot was used to maintain the growth of T cells and counter the TME. Grosskopf et al.^184^ developed an advanced version of a scaffold platform in which they co‐delivered CAR‐T cells and stimulatory cytokines through in‐situ formed polymer‐nanoparticle (PNP) hydrogels. The cytokine encapsulated in the hydrogel‐embedded nanoparticles was released over time in the surrounding hydrogel network where CAR T cells were loaded. This design allowed CAR‐T cells to persist and activate for prolonged periods of time after local injection in the NSG mice bearing a subcutaneously implanted human solid tumor of medulloblastoma. The sustained exposure of T cells and their stimulatory cytokine secretion also led to clearance of distal tumors in 100% of mice treated with CAR‐T cells loaded in the depot as compared to 80% tumor‐free survival in the group receiving bolus CAR‐T cell treatment. Additionally, depots loaded with stem cells have also been reported for brain tumor treatment.^185^ Stem cells like MSCs and NSCs that are modified to secrete cytotoxic agents have been administered locally to the brain tumor through various biomaterial scaffolds to achieve extended persistence. Kauer et al.^186^ prepared a synthetic extracellular matrix hydrogel to encapsulate NSCs and MSCs. In a resection model of orthotopic GBM, 100% of mice treated with the hydrogels loaded with NSCs modified to secrete TRAIL were alive, while all mice injected with NSC suspension died by 42 days after treatment. Many similar strategies have been developed over the years, collectively demonstrating the therapeutic benefits of locoregional delivery using biomaterial depots/scaffolds in various preclinical brain tumor models. With the advent of injectable formulations, these strategies could be feasibly adopted in clinical settings for improving the distribution and persistence of therapeutic cells, offering new opportunities to promote the translation of cell therapies against brain cancer.

### 5.3 BBB opening to increase delivery efficiency

Apart from modifying cells or using various routes of administration, many other approaches have been investigated to promote therapeutic cell infiltration into the brain. One promising strategy is to transiently elicit the BBB opening. Sabbagh et al.^187^ demonstrated the potential of low‐intensity pulsed ultrasound (LIPU)‐mediated transient BBB opening for increasing the effectiveness of immunotherapies, such as EGFRvIII‐CAR T cells and anti‐PD‐1 antibodies. In the GL261 orthotopically implanted GBM murine model, LIPU increased the delivery of CAR T cells and anti‐PD‐1 antibody substantially following intravenous injection. This combination treatment significantly improved median survival compared to either CAR T cells or antibody alone. CAR T cells plus LIPU increased median survival by more than 129% compared to CAR T cells alone. Furthermore, intravenous administration of CXCL10‐secreting APCs with LIPU significantly enhanced their accumulation in the brain, facilitated intratumoral infiltration of CD8^+^ T cells, and increased survival rates.^187^ Similarly, Alkins et al.^188^ used focused ultrasound (FUS) to improve the delivery of CAR NK‐92 cells targeting HER2. The longitudinal treatment with NK‐92 cells targeting HER2 and FUS resulted in long‐term survival in a HER2‐overexpressing brain tumor‐bearing mice. The safety of the ultrasound approach has been demonstrated in human clinical studies.^189^ The transient BBB opening strategy with ultrasound has recently been examined in clinical trials with both transcranial and implantable FUS devices for treating recurrent GBM, in which TMZ (NCT04614493), bevacizumab (NCT04446416), and albumin‐bound paclitaxel and carboplatin (NCT04528680) are used as therapeutic drugs. Importantly, a phase 2a trial involving an implantable ultrasound device (Sonocloud‐9) with concomitant balstilimab (anti‐CTLA‐4 antibody), botensilimab (anti‐PD‐1 antibody), and liposomal doxorubicin is actively ongoing for newly diagnosed GBM patients (NCT05864534). With increasing clinical trials demonstrating the usefulness of immunotherapies combined with ultrasound, it is expected that the ultrasound‐mediated BBB opening strategy could be rapidly translated into clinical studies for enhancing cell therapy delivery.

### 5.4 Material‐based cell‐surface engineering

Material‐based cell surface engineering is another promising approach for improving the therapeutic effect of cells. This approach offers several advantages, including enhancement of cell trafficking, targeted delivery of therapeutic molecules to desired tissues, and modulation of the immunophenotype of carrier cells. One promising approach in cell surface engineering for cancer immunotherapy is the use of disk‐shaped microparticles, termed “backpacks” which attach to the cell surface without being internalized due to their high aspect ratio.^190^ A recent study demonstrated that integrin‐mediated attachment of backpacks activates neutrophils without the use of any neutrophil activators, based on the fact that neutrophils are activated through frustrated phagocytosis when physically attached to macroscopic surfaces.^191^ The adoptive transfer of backpack‐activated N1 neutrophils systemically elicited potent anti‐tumor immune responses through activation of immune cells and inhibited tumor progression in murine models of breast cancer and melanoma. Furthermore, neutrophil‐backpack treatment showed a synergistically improved therapeutic effect when combined with immune checkpoint blockade.^191^ The therapeutic potential of backpack‐activated neutrophils against GBM was also investigated. The combined administration of neutrophil‐backpacks and anti‐PD‐1 antibody induced T cell‐mediated anti‐tumor immune responses systemically and significantly augmented the therapeutic efficacy of the anti‐PD‐1 antibody in an orthotopically GL261 GBM model. Of note, such backpack attachment does not affect neutrophil migration into the brain after intravenous injection.^192^ Neutrophils offer unique advantages and challenges to clinical translation due to their intrinsic biology. MHC‐independent neutrophil activity provides a choice of a wider donor population for allogeneic transfer. However, cryopreservation of neutrophils for a long duration is not advised because of their short lifespan. Granulocyte transfusion therapies are generally carried out within 6 h of allogeneic cell harvest.^193^ Importantly, the backpack technique can polarize neutrophils to an N1‐activated phenotype within <2 h without genetic modification or long‐term biochemical stimulation, indicating the possibility of modification of neutrophils and their infusion into the patients within the recommended six‐hour timeframe. This material‐based surface engineering approach offers the possibility of developing neutrophil‐based GBM therapies. Additionally, backpack‐based cell surface engineering can be applied for cancer immunotherapy with other types of immune cells, including NK cells, B cells, monocytes, and macrophages.^34^, ^129^, ^194^, ^195^, ^196^ With further developments, it is expected that the backpack‐mediated approach could be clinically translated for treating brain cancer using diverse types of cells.

## 6 CONCLUSIONS

Clinical efficacy of the current standard therapy against brain tumors, particularly GBM, continues to show poor clinical efficacy. Despite being the standard for over 30 years, there has been little meaningful improvement in patient outcomes. Cells represent a promising therapeutic modality in transforming the challenging landscape of treating brain tumors. Our article discusses the history of adoptive cell transfer for brain tumor therapy, critically analyzes their clinical landscape, and identifies key challenges from past clinical experience. We also highlight recent promising preclinical studies to address such challenges. The number of clinical trials and preclinical studies for brain tumor treatment using cell therapies has been increasing, particularly following the clinical success of CAR‐T cell therapies. Several clinical challenges have emerged from clinical trials investigating various types of cell therapies against brain cancer, which include intricate tumor heterogeneity, poor delivery efficiency, and exhaustion of injected cells in the immunosuppressive TME. Significant efforts are being made to address these clinical challenges through the development of new genetic modifications, material‐based engineering approaches, and optimized locoregional delivery methods. Given the success of cell therapies in the clinic for other cancer types and the ongoing development in preclinical and clinical settings, cell therapy products are expected to become viable treatment options for brain tumor patients. Continued research and collaborations will be essential for refining these therapies and realizing their full potential in the fight against this devastating disease.

## AUTHOR CONTRIBUTIONS

**Tatsuya Fukuta:** Conceptualization; investigation; methodology; formal analysis; data curation; visualization; writing – original draft; writing – review and editing. **Suyog Shaha:** Conceptualization; investigation; methodology; visualization; formal analysis; data curation; writing – original draft; writing – review and editing. **Andres da Silva‐Candal:** Methodology; formal analysis. **Zongmin Zhao:** Writing – review and editing. **Samir Mitragotri:** Conceptualization; funding acquisition; writing – review and editing.

## CONFLICT OF INTEREST STATEMENT

Samir Mitragotri is an inventor on patent applications that relate to the technologies discussed herein. These patents are owned and under the management of Harvard University. No competing interests are reported by the other authors.

## Data Availability

All necessary data are available in the manuscript.
